# Natural Phytocompounds from Common Indian Spices for Identification of Three Potential Inhibitors of Breast Cancer: A Molecular Modelling Approach

**DOI:** 10.3390/molecules27196590

**Published:** 2022-10-05

**Authors:** Samik Hazra, Anindya Sundar Ray, Chowdhury Habibur Rahaman

**Affiliations:** 1 Ethnopharmacology Laboratory, Department of Botany, Visva-Bharati, Santiniketan 731235, West Bengal, India; 2 Department of Animal Science, Kazi Nazrul University, Asansol 713340, West Bengal, India

**Keywords:** breast cancer, Indian spices, molecular docking, drug likeliness

## Abstract

Breast cancer is the second most common cancer-related cause of death for women throughout the globe. In spite of some effective measures, the main concerns with traditional anti-cancer chemotherapy are its low bioavailability, physical side effects, acquired resistance of cancer cells and non-specific targeting. Now researchers have taken the initiative to establish natural product-based therapy methods and to identify viable hits for future lead optimization in the development of breast cancer medication. Our study aims to identify the potent phytocompounds from five very popular Indian spices (*Zingiber officinale* Roscoe, *Cuminum cyminum* L., *Piper nigrum* L., *Curcuma longa* L., and *Allium sativum* L.). From these spices, a total of 200 phytocompounds were identified and screened against three target genes, namely, cyclin-dependent kinase 8 (CDK 8), progesterone receptor (PR) and epidermal growth factor receptor (EGFR), through structure-based virtual screening using iGEMDOCK 2.1 software. Based on the binding affinity score, the top three phytocompounds against each target protein (cynaroside (−149.66 Kcal/mol), apigetrin (−139.527 Kcal/mol) and curcumin (−138.149 Kcal/mol) against CDK8; apigetrin (−123.298 Kcal/mol), cynaroside (−118.635 Kcal/mol) and xyloglucan (−113.788 Kcal/mol) against PR; cynaroside (−119.18 Kcal/mol), apigetrin (−105.185 Kcal/mol) and xyloglucan (−105.106 Kcal/mol) against EGFR) were selected. Apigetrin, cynaroside, curcumin, and xyloglucan were finally identified for further docking analysis with the respective three target proteins. Autodock 4.2 was applied to screen the optimal binding position and to assess the relative intensity of binding interactions. In addition, the ADME/T property checks and bioactivity scores analysis of were performed to understand the suitability of these four phytocompounds to be potential candidates for developing effective and non-toxic anticancer agents. Based on this in silico analysis, we believe this study could contribute to current efforts to develop new drugs for treating breast cancer.

## 1. Introduction

An increased incidence in recent years and its influence on various physical, mental, and social aspects of human existence have elevated cancer to a major challenge of the century. Cancer is a broad word that encompasses a wide range of diseases that can affect any region of the body. One of the hallmarks of cancer is the emergence of abnormal cells that expand beyond their usual boundaries, allowing them to invade neighbouring parts of the body and migrate to other organs (metastasis) [1]. In 2020, an estimated 19.3 million new cancer cases (18.1 million excluding nonmelanoma skin cancer) with almost 10.0 million cancer deaths (9.9 million excluding nonmelanoma skin cancer) were recorded [2]. On the other hand, breast cancer has surpassed lung cancer as the world’s most prevalent cancer type, with 7.8 million women diagnosed with the disease in the previous five years by the end of 2020 [3]. Breast cancer is metastatic cancer that can spread to other organs, including the bone, liver, lung, and brain, which is primarily accountable for its incurability. As the data indicates, it is one of the dominant causes of death in many countries. In India, breast cancer is one of the most common cancers, accounting for more than 27% of all cancer patients. Breast cancer is diagnosed in one out of every 22 women in cities, and one out of every 60 women in rural provinces [4]. Despite the fact that the exact cause of breast cancer is unclear, the associated risk factors have been discovered. Aging, a family history of breast cancer, particular alterations in the breast (s), genetic changes, productivity and menopausal history, lack of physical activity, alcohol abuse, obesity, diet, race, and chest radiation therapy are a few among the numerous risk factors that are associated with breast cancer [5].

Despite incredible progress in diagnosis and development of several targeted therapeutic methods, breast cancer is still the leading cause of death caused by cancer in women worldwide [6]. The existing therapeutic approaches are associated with high cost, significant toxicity, therapeutic resistance, low efficacy, and therapy related morbidity [5]. Recent studies have reported that owing to their cancer preventive potential natural products will pave a way for more effective, nontoxic, non-endocrine, and therapeutic approaches for anticancer remedies [7]. A deeper understanding of the molecular mechanisms involved in breast cancer progression and identification of new active compounds should be helpful in developing more effective treatments for breast cancer. Equipped with modern technology and ideas, global pharmaceutical companies have started to rediscover medicinal plants as a possible source of novel anticarcinogenic drug candidates and have renewed their strategies favouring natural product-based drug discovery [8].

Pharmacology has a long history of scientists who have been able to create qualitative or semi-quantitative relationships between molecular structure and activity in cerebro. They have consistently employed standard pharmacology methods such as in vivo and in vitro models to explore these theories. However, during the last decade, we have witnessed a significant increase in the development and application of computational (in silico) methodologies in the field of drug discovery [9]. Traditional methods are anticipated to take roughly 12 years and cost around 2.7 billion dollars on average for the development of a new drug [10]. Computational prediction tools have been widely employed to enhance the effectiveness of drug discovery because they allow researchers to test a large number of compounds in a short amount of time without incurring high costs or sacrificing animals. This can provide preliminary data for further in vitro and in vivo studies, which improves the study’s success rate [11,12]. The number of target proteins with a known three-dimensional structure is increasing rapidly due to the advancements in techniques for structure determination, such as high-throughput X-ray crystallography [13]. From hit identification through lead optimization and beyond, molecular modelling has been an essential part of many drug-development programs [14]. The study of the chemical make-up of a drug, its interactions with putative disease-relevant targets, and projections of its ADMET characteristics are all included in molecular modelling [15]. The application of molecular modelling is very broad and multifaceted [15]. One crucial technique of molecular modelling is the molecular docking study which involves docking of small molecules to protein binding sites [16]. It was developed in the early 1980s [17] and is still a very active field of research today [16]. It may be used at many phases of the drug design process including, screening of millions of compounds in an affordable time, anticipation of the binding mode of ligands, identification and characterization of potent ligands, and the quantification of the free energy of protein–ligand binding (∆G) [15,18]. Indeed, a number of modern chemotherapies have been significantly influenced by the structure-based design and screening techniques [15]. One of the first implementations of docking was for HIV drug discovery by DesJarlais and Dixon [19]. Additionally, docking can also help in drug metabolism analysis [20,21]. In our study we have employed a structure-based virtual screening approach to screen novel bioactive molecules from common Indian spices to identify potential inhibitors against certain breast cancer targets. There is no previous study that has been conducted to evaluate the anticancer potential of the phytocompounds trapped inside the studied spice plants.

Targeted therapy is a type of cancer treatment in which drugs are used to target specific genes and proteins in cancer cells that aid in the survival and growth of cancer cells. Targeted therapy has the ability to alter the environment in which cancer cells thrive or can destroy cancer-causing cells. Subsequent identification and characterization of several target proteins that regulate cell growth, differentiation, motility, and apoptosis has ushered in a new age of cancer therapy [22]. Proteins including progesterone receptor (PR), epidermal growth factor receptor (EGFR), mechanistic target of rapamycin (mTOR), p53R2, cytotoxic T-lymphocyte–associated antigen 4 (CTLA-4), cyclin dependent kinase-(CDK8), etc. can be used as therapeutic targets to stop cancer from progressing [23,24]. PRs are members of the steroid hormone receptor (SR) subfamily of nuclear receptors that are ligand-activated transcription factors. PRs are important transcriptional regulators, but they also activate signal transduction pathways, including some that are associated with pro-proliferative signalling in the breast [25]. One of the earliest known major targets of the emerging anticancer drugs is the EGFR. Triple-negative breast cancer (TNBC) and inflammatory breast cancer (IBC) both overexpress EGFR in around half of the cases. EGFR inhibitors have therefore been tested in various studies for the treatment of breast cancer [26]. CDK8 is a nuclear serine–threonine kinase that functions as a transcriptional regulator that cooperates with several transcription factors [27]. CDK8 expression was found to be upregulated in breast cancers and linked to tumour progression [28].

In the present study, we have chosen five Indian spice-yielding plants: *Zingiber officinale* Roscoe (ginger), *Cuminum cyminum* L. (cumin), *Piper nigrum* L. (black pepper), *Curcuma longa* L. (turmeric), and *Allium sativum* L. (garlic) as their broad-spectrum therapeutic properties are experimentally proved and well documented in various literature [29]. From these plants, a total of 200 phytocompounds (**30** for *Zingiber officinale* Roscoe; **41** for *Cuminum cyminum* L.; **41** for *Piper nigrum* L.; **42** for *Curcuma longa* L., and **46** for *Allium sativum* L.) were screened against various breast cancer protein targets (i.e., CDK8, PR and EFGR). The primary objective of this study is to identify bioactive compounds by screening a large number of phytochemicals by employing a bioinformatic approach in order to develop an effective breast cancer therapy. For comparative evaluation and to highlight the extreme effectiveness of the natural products, marketed anti-cancer standard drugs were also included in the present study. Our study also imbibes the drug likeliness and toxicity profiles of the selected phytocompounds for assessing qualitatively the chances of the selected compounds to become oral drugs with respect to their bioavailability and to understand their safety and efficacy as potent drug candidates.

## 2. Result and Discussion

Due to the advances in genetic testing, immunotherapy, and other areas, survival rates for breast cancer have improved in recent decades. Despite this, breast cancer remains a serious public health concern, with it receiving the top priority in medical research [30]. The primary goal of our research is to identify possible breast cancer inhibitors by employing various computational tools. An attempt was made to test different phytochemicals against multiple proteins associated with breast cancer progression and regarded as potential drug targets.

### 2.1. Molecular Docking

The molecular docking technique is used to mimic the interaction between a ligand and a protein at the atomic level, allowing us to predict the behaviour of the ligands in target protein binding sites. The docking technique consists of two main steps: predicting the ligand structure as well as its location and orientation inside these sites (known as pose) and estimating the binding affinity [31]. A total of 153 phytocompounds were analysed for their activities against the target proteins. To determine the binding affinities of 153 phytocompounds to 3 target proteins, docking studies were performed using the iGEMDOCK 2.1 software. The binding interaction and conformation of each phytocompound with each target protein were predicted and rated on the basis of the lowest energy and total binding energy, respectively. The docking score of the ligands against the receptors used in the study are mentioned in Supplementary File Table S3. Among the 153 compounds the top three phytocompounds against each target proteins were selected and docking analysis was then carried out with all the target proteins through the AutoDock 4.2 programme. Based on the higher binding affinity, cymaroside (−149.66 Kcal/mol), apigetrin (−139.527 Kcal/mol) and curcumin (−138.149 Kcal/mol) against CDK8 were selected. Against PR the top performing drug candidates were apigetrin (−123.298 Kcal/mol), cymaroside (−118.635 Kcal/mol) and xyloglucan (−113.788 Kcal/mol). Similarly, against EGFR three phytocompounds, namely cynaroside (−119.18 Kcal/mol), apigetrin (−105.185 Kcal/mol) and xyloglucan (−105.106 Kcal/mol) were selected based on their binding affinity. To determine the relative strengths of the binding interactions of the best identified phytocompounds, the Autodock (v4.2) software was used to search for its best binding pose and to compare the binding affinity with each target protein. Autodock maintained by Scripps Research, specifically the Center for Computational Structural Biology (CCSB) located in the Torrey Mesa region of La Jolla, California, USA.

### 2.2. Screening of Drug Candidates against CDK8

**Figure 1:**
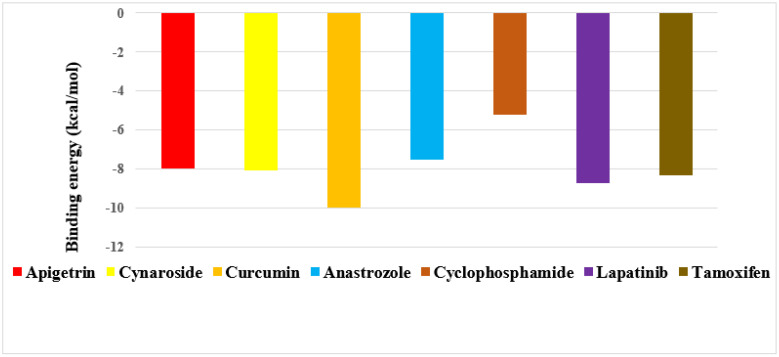
Graphical representation of the binding energy (−kcal/mol) of the top three phytochemicals (apigetrin, cynaroside and curcumin) and standard drugs (anastrozole, cyclophosphamide, lapatinib and tamoxifen) in complexation with CDK8.

**Figure 2:**
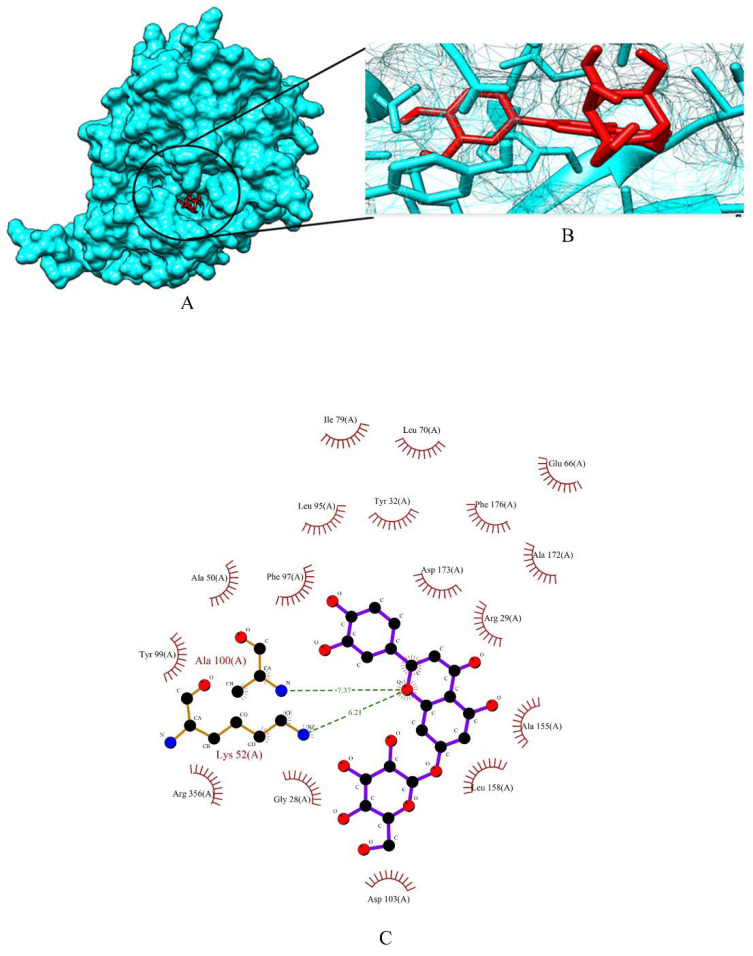
(**A**) Surface view of cynaroside -CDK8 binding complex. (**B**) Binding pose of cynaroside. (**C**) Visualization of the docked complex with ligplot^+^ shows the binding interaction of cynaroside with CDK8. Two hydrogen bond interactions (green dotted lines) were formed between cynaroside and two amino acid residues (Lys 52, Ala 100) of target protein CDK8.

**Figure 3:**
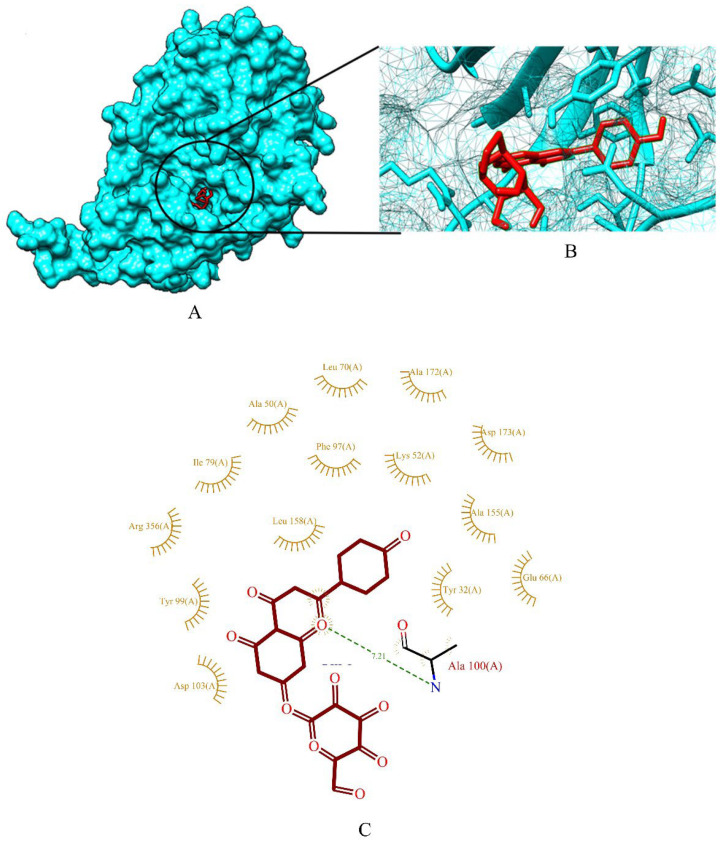
(**A**) Surface view of apigetrin-CDK8 binding complex. (**B**) Binding pose of apigetrin. (**C**) Visualization of the docked complex with ligplot^+^ shows the binding interaction of apigetrin with CDK8. One hydrogen bond interaction (green dotted line) was formed between apigetrin and one amino acid residue (Ala 100) of target protein of CDK8.

**Figure 4:**
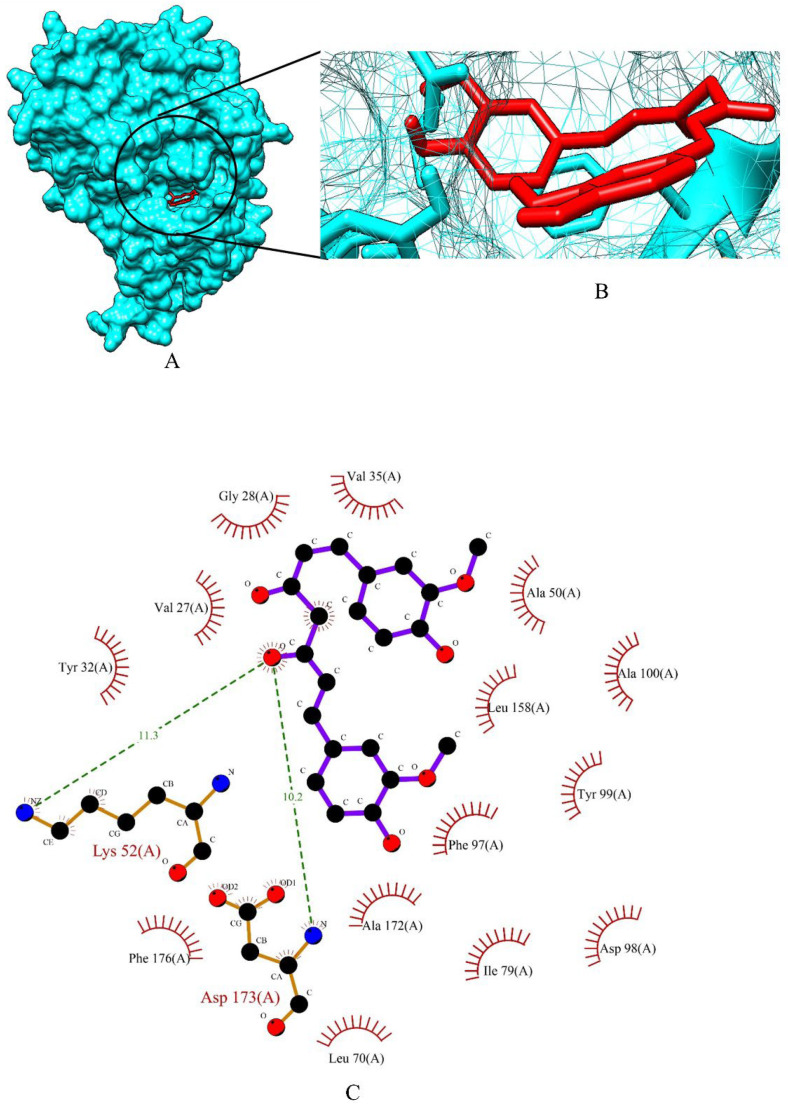
(**A**) Surface view of curcumin-CDK8 binding complex. (**B**) Binding pose of curcumin. (**C**) Visualization of the docked complex with ligplot^+^ shows the binding interaction of curcumin with CDK8. Two hydrogen bond interactions (green dotted lines) were formed between curcumin and two amino acid residues (Lys 52, Asp 137) of target protein CDK8.

The binding energy of three prime phytocompounds (cynaroside, apigetrin and curcumin) were between −7.97 kcal/mol to −9.97 kcal/mol, while the binding energy of standard drugs (anastrozole, cyclophosphamide, lapatinib and tamoxifen) were between −5.23 kcal/mol to −8.74 kcal/mol. The graphical representation of the binding energy (kcal/mol) of the top three phytochemicals and standard drugs in complexation with the CDK8 is shown in Figure 1. Figure 2 and Figure 3 exhibited hydrogen bond interactions of the cymaroside and apigetrin with the target protein CDK8. respectively. The ligand curcumin with the lowest binding affinity was found to form two bonds involving two amino acid residues (Lys 52, Asp 137) of the CDK8 protein (Figure 4A–C). Curcumin (−9.97 kcal/mol) was found to outperform all the standard drugs and cynaroside (8.08 kcal/mol) has shown better binding than the standard drug anastrozole (−7.51 kcal/mol) and cyclophosphamide (−5.23 kcal/mol).

### 2.3. Screening of Drug Candidates against PR

**Figure 5:**
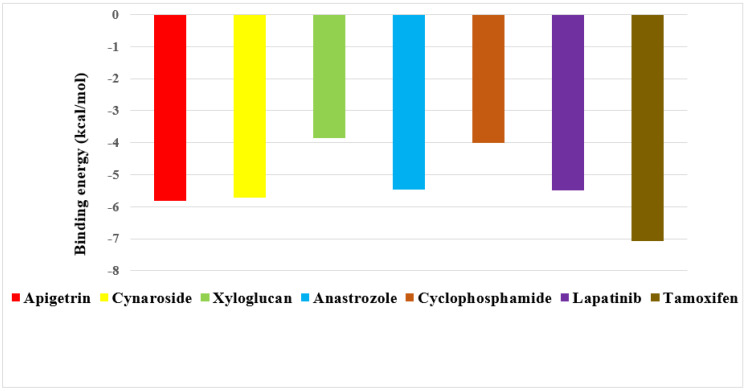
Graphical representation of the binding energy (−kcal/mol) of the top three phytochemicals (apigetrin, cynaroside and xyloglucan) and standard drugs (anastrozole, cyclophosphamide, lapatinib and tamoxifen) in complexation with PR.

**Figure 6:**
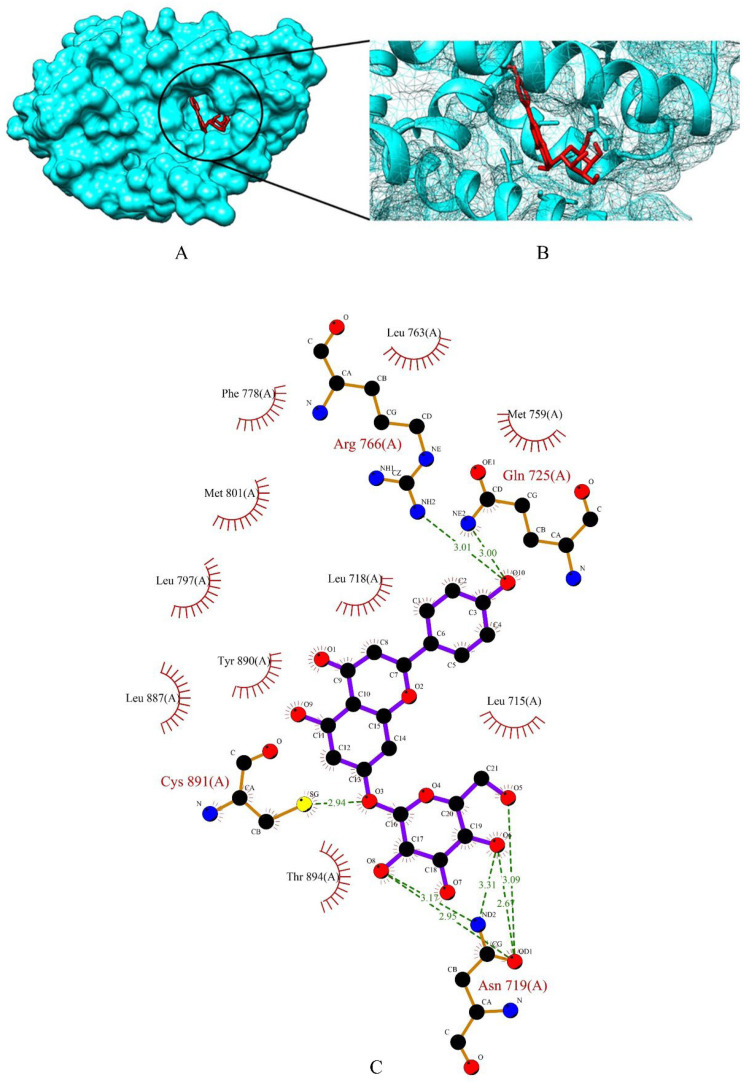
(**A**) Surface view of apigetrin-PR binding complex. (**B**) Binding pose of apigetrin. (**C**) Visualization of the docked complex with ligplot^+^ shows the binding interaction of apigetrin with PR. Eight hydrogen bond interactions (green dotted lines) were formed between apigetrin and four amino acid residues (Asn 719, Gln 725, Arg 766, Cys 891) of target protein PR.

**Figure 7:**
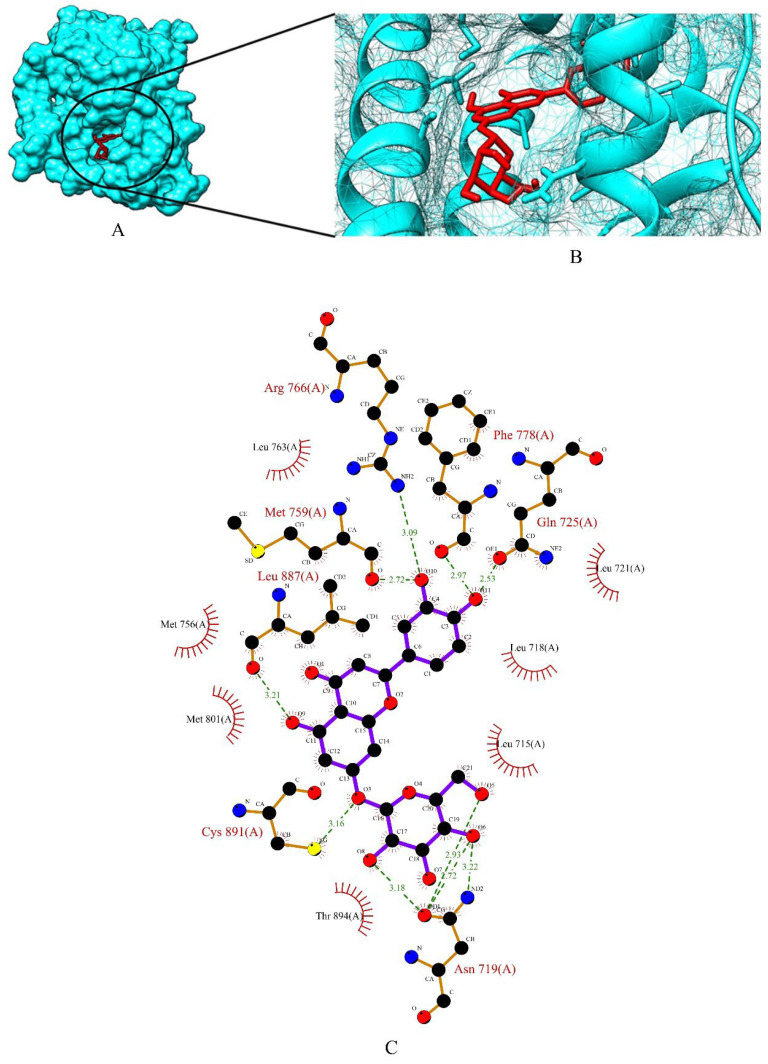
(**A**) Surface view of cynaroside-PR binding complex. (**B**) Binding pose of cynaroside. (**C**) Visualization of the docked complex with ligplot^+^ shows the binding interaction of cynaroside with PR. Ten hydrogen bond interactions (green dotted lines) were formed between cynaroside and seven amino acid residues (Asn 719, Gln 725, Met 759, Arg 766, Phe 778, Leu 887, Cys 891) of target protein PR.

**Figure 8:**
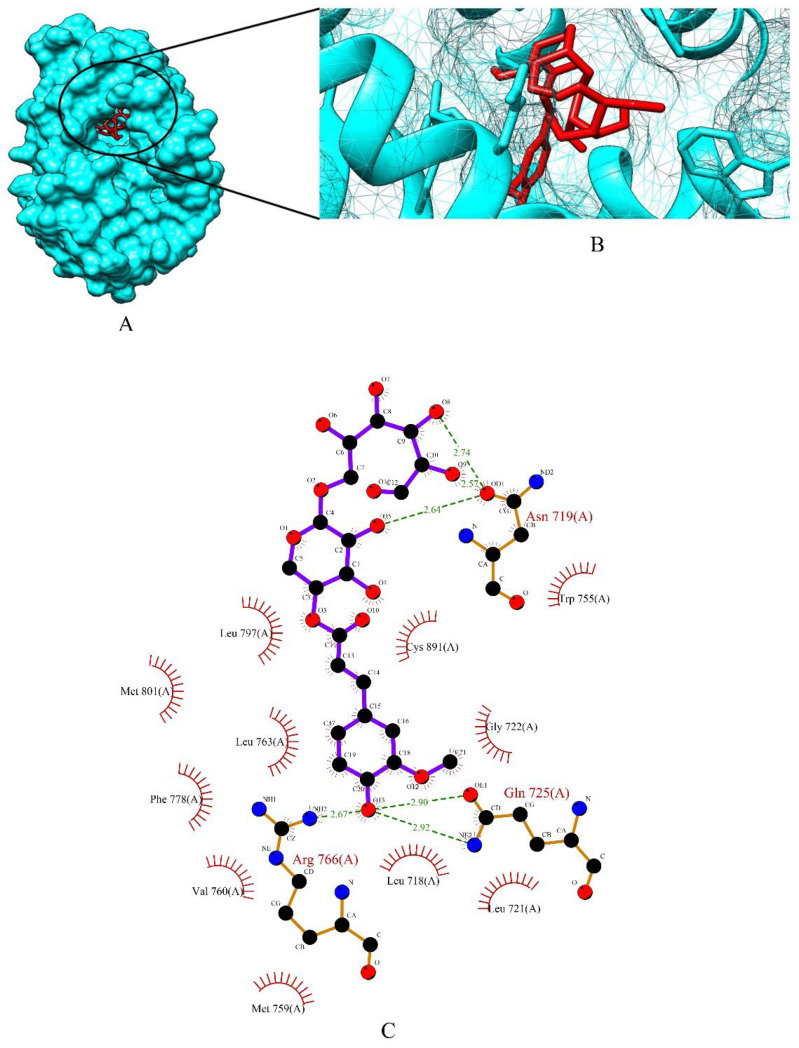
(**A**) Surface view of xyloglucan-PR binding complex. (**B**) Binding pose of xyloglucan. (**C**) Visualization of the docked complex with ligplot^+^ shows the binding interaction of xyloglucan with PR. Six hydrogen bond interactions (green dotted lines) were formed between xyloglucan and three amino acid residues (Asn 719, Gln 725, Arg 766) of target protein PR.

The top three phytochemicals viz apigetrin, cynaroside and xyloglucan had the lowest binding affinity of −5.83 kcal/mol, −5.71 kcal/mol and −3.86 kcal/mol, respectively. The binding energy of the standard drugs (anastrozole, cyclophosphamide, lapatinib and tamoxifen) were in the range of −4.00 kcal/mol to −7.07 kcal/mol. The graphical representation of the binding energy (-kcal/mol) of the top three phytochemicals and standard drugs in complexation with the PR is shown in Figure 5. Apigetrin (−5.83 kcal/mol) with the lowest affinity among the phytocompounds was found to form eight hydrogen bonds involving four amino acid residues (Asn 719, Gln 725, Arg 766 and Cys 891) of the receptor protein (Figure 6A–C). The binding energy of apigetrin (−5.83 kcal/mol) and cynaroside (−5.71 kcal/mol) were more than the standard drug lapatinib (−5.5 kcal/mol) anastrozole (−5.46 kcal/mol) and cyclophosphamide (−4.0 kcal/mol). Among all the seven ligands tamoxifen was found to have highest binding energy of −7.07 kcal/mol. Figure 7 and Figure 8 shows hydrogen bond interactions of the cynaroside-PR and xyloglucan-PR docked complex respectively.

### 2.4. Screening of Drug Candidates against EGFR

**Figure 9:**
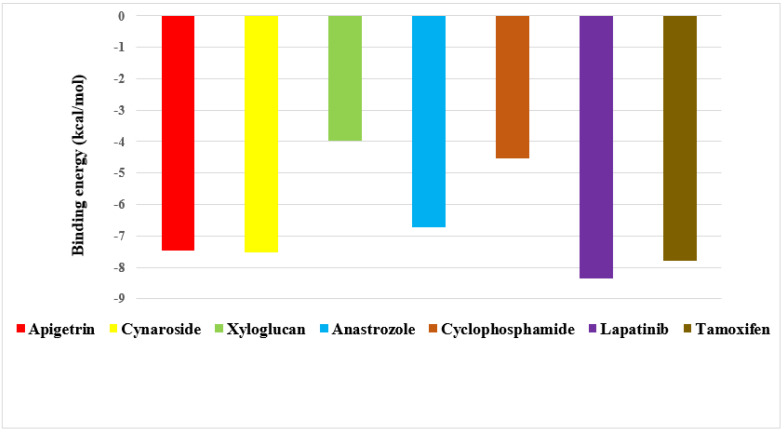
Graphical representation of the binding energy (−kcal/mol) of the top three phytochemicals (apigetrin, cynaroside and xyloglucan) and standard drugs (anastrozole, cyclophosphamide, lapatinib and tamoxifen) in complexation with EGFR.

**Figure 10:**
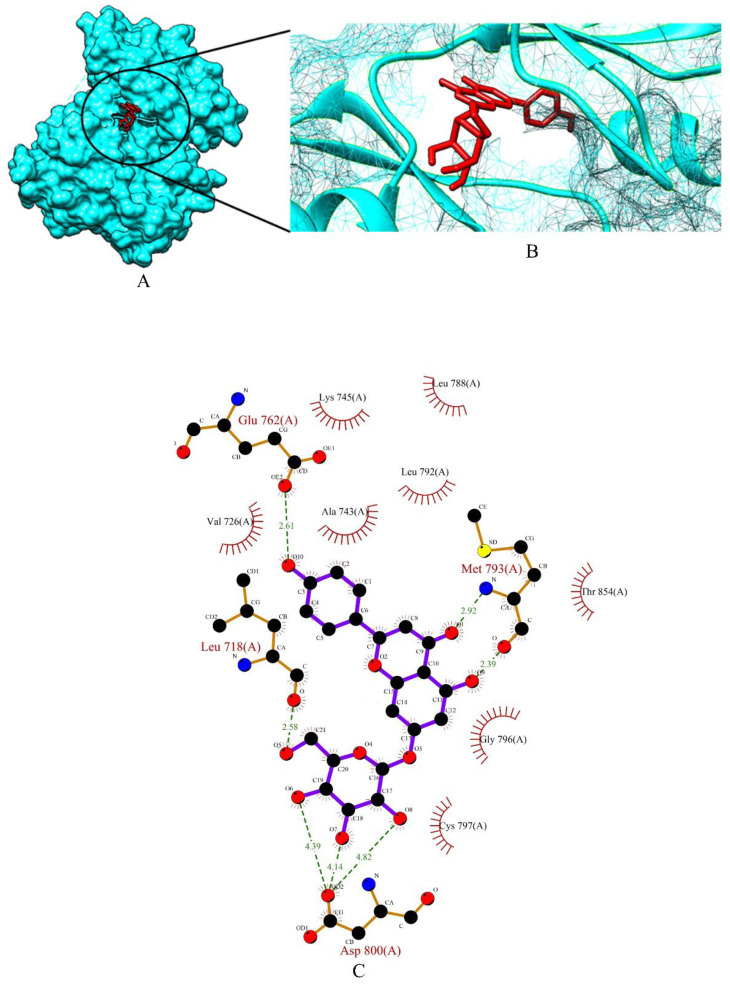
(**A**) Surface view of apigetrin-EGFR binding complex. (**B**) Binding pose of apigetrin. (**C**) Visualization of the docked complex with ligplot^+^ shows the binding interaction of apigetrin with EGFR. Seven hydrogen bond interactions (green dotted lines) were formed between apigetrin and four amino acid residues (Leu 718, Glu 762, Met 793, Asp 800) of target protein EGFR.

**Figure 11:**
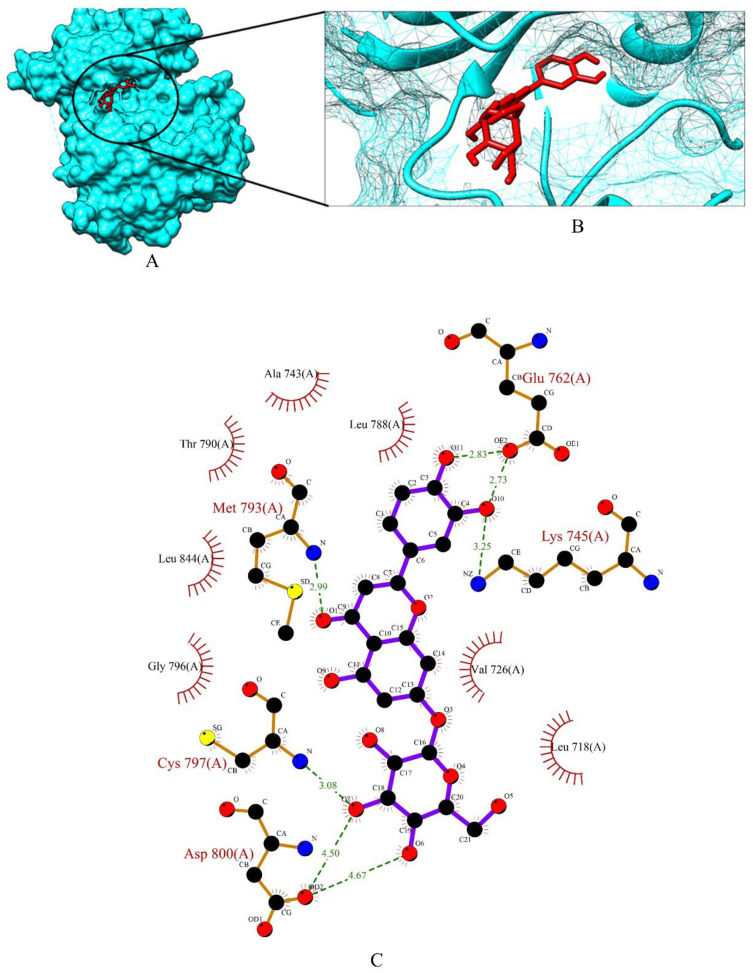
(**A**) Surface view of cynaroside-EGFR binding complex. (**B**) Binding pose of cynaroside. (**C**) Visualization of the docked complex with ligplot^+^ shows the binding interaction of cynaroside with EGFR. Seven hydrogen bond interactions (green dotted lines) were formed between cynaroside and four amino acid residues (Lys 745, Glu 762, Met 793, Cys 979, Asp 800) of target protein EGFR.

**Figure 12:**
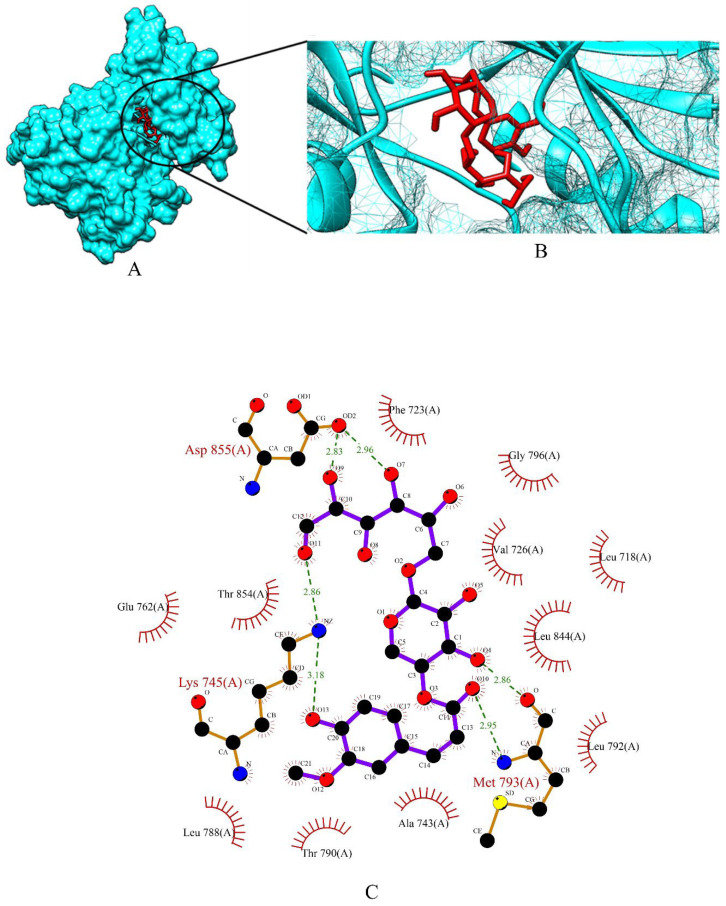
(**A**) Surface view of xyloglucan-EGFR binding complex. (**B**) Binding pose of xyloglucan. (**C**) Visualization of the docked complex with ligplot^+^ shows the binding interaction of xyloglucan with EGFR. Six hydrogen bond interactions (green dotted lines) were formed by xyloglucan involving three amino acid residues (Lys 745, Met 793, Asp 855) of the target protein.

Apigetrin, cynaroside and xyloglucan chosen from a total of 153 molecules have shown binding affinity energy values of −7.47 kcal/mol, −7.51 kcal/mol, and −3.96 kcal/mol for EGFR. The binding affinity range of the standard drugs (anastrozole, cyclophosphamide, Lapatinib and tamoxifen) was between −4.53 kcal/mol to −8.53 kcal/mol. The graphical representation of the binding energy (-kcal/mol) of the top three phytochemicals and standard drugs in complexation with the EGFR receptor is shown in Figure 9. Cynaroside (−7.51 kcal/mol) and apigetrin (−7.47 kcal/mol) were found to have lower binding energy than the standard drugs cyclophosphamide (−4.53 kcal/mol) and anastrozole (−6.72 kcal/mol). The binding energy value of tamoxifen (−7.79 kcal/mol) is very similar with the binding energy values of cynaroside and apigetrin with just a difference of 0.28 and 0.32, respectively. The phytocompounds viz. apigetrin, cymaroside and xyloglucan also formed hydrogen-bond interactions with the residues of targeted protein EGFR, as shown by their corresponding 3D interaction models in Figure 10, Figure 11 and Figure 12, respectively.

Several approaches to treat breast cancer include surgery, chemotherapy, immunotherapy, gene therapy, and protein therapy [32]. As we have mentioned earlier, the costs of conventional cancer-therapy are too expensive, making it difficult for people in low- and middle-income countries to afford their medical care. On the other hand, the majority of anticancer medications have a number of side effects in addition to the issue of drug resistance. Due to their structural variety, few side effects, high bioavailability, and various target activities, plant-based bioactive extracts and phytochemicals are currently gaining popularity [33]. Rational drug design employing in silico approach involves the usage of molecular modelling techniques like pharmacophore modelling, virtual screening, and molecular docking to predict the interaction of molecules with the drug targets to develop effective drug candidates [34].

The results of our study indicate that Indian spice plants are rich in phytochemicals that can inhibit specific breast cancer targets. In accordance with the scoring function of the iGEMDOCK among 153 molecules, the top four drug candidates namely, apigetrin, cynaroside, curcumin and xyloglucan were selected. Our findings are in agreement with the earlier research findings that the lower binding energy score gives greater protein-ligand binding stability [35]. When these phytocompounds were subjected to further docking study using Autodock 4.2, they were evaluated based on their free energy of binding (∆G). The free energy of binding (∆G), which is equal to the free energy of the protein–ligand complex minus the free energies of the protein and the ligand in their unbound states, is used to quantitatively measure the degree of spontaneity and strength of protein–ligand binding [15]. In our study, among these four compounds, curcumin, apigetrin, and cynaroside were found to be the potential inhibitors of CDK8, PR, and EGFR, respectively as they have lowest binding energy and higher binding affinity to the binding sites of the targets. Four marketed oral breast cancer drugs i.e., anastrozole, cyclophosphamide, lapatinib and tamoxifen were also subjected to molecular docking as standard for comparative evaluation. It was found that the four best identified phytocompounds most of the time showed greater binding affinity to the target proteins comparing with the standard drugs. As per example, during screening of drug candidates against CDK8, it was found that curcumin (−9.97 kcal/mol) has the lowest binding energy among all the seven ligands, including the standard drugs. While cynaroside (−8.08 kcal/mol) was found to have greater binding affinity than anastrozole (−7.51 kcal/mol) and cyclophosphamide (−5.23 kcal/mol). Similarly, apigetrin (−5.83 kcal/mol) and cynaroside (−5.71 kcal/mol) were found to have lower binding energies than the conventional drugs lapatinib (−5.5 kcal/mol), anastrozole (−5.46 kcal/mol), and cyclophosphamide (−4.0 kcal/mol) during their docking against PR. A similar pattern is evident during docking of ligands against EGFR. Cynaroside (−7.51 kcal/mol) and apigetrin (−7.47 kcal/mol) were found to have lower binding energy than the standard drug cyclophosphamide (−4.53 kcal/mol) and anastrozole (−6.72 kcal/mol). Various experimental studies have also confirmed the anti-carcinogenic properties of these three compounds. Kim et al., 2020 elucidated the anticarcinogenic property of apigetrin. The study revealed that apigetrin had induced the extrinsic pathway of apoptosis, and also prompted the autophagy, cell cycle arrest in AGS human gastric cancer cell line through the PI3K/AKT/mTOR pathway [36]. Several experiments have also revealed that, cynaroside has varying degrees of anticancer activity in non-small cell lung cancer, liver cancer, oesophageal cancer, gastric cancer, and cervical cancer. A study performed by Ji et al., 2021 reported that the cynarosides inhibited the MET/AKT/mTOR signalling pathway, which regulates a variety of biological processes such as cell proliferation, apoptosis, autophagy, invasion, and tumorigenesis, by lowering the phosphorylation levels of AKT, mTOR, and P70S6K [37]. Curcumin has been demonstrated in several studies to have anticancer properties as it decreases cancer cell growth and metastasis by arresting cell cycle progression and apoptosis. Curcumin also prevents breast cancer stem cells (BCSC), an important factor that influences cancer recurrence, from proliferating [38]. These experimental studies validate our in-silico screening of potential inhibitors of breast cancer. The analogues of these highly effective natural compounds can be designed using pharmacophore modelling and drug design. To confirm our in-silico study, in vitro and in vivo experiments must be carried out, and if the results are in agreement with our findings, the phytochemicals can be chemically synthesised and will be marketed as anticancer therapy.

### 2.5. Drug Likeness and Toxicity Prediction

Lipinski and co-workers theoretically evaluated drug likeness properties of drug candidates for the first time in 1997 when they published the Rule of Five (Ro5) [39]. Drug likeness can be defined as a perplexing equilibrium of various molecular characteristics and structural traits that determines whether a given compound is a drug or nondrug. These properties, mainly lipophilicity, electronic distribution, hydrogen bonding characteristics, molecule size and flexibility, and presence of various pharmacophoric features that influence the behaviour of a molecule in a living organism which include characteristics such as transport, affinity to proteins, reactivity, toxicity, metabolic stability, and many others. To predict the drug likeness of a drug, this rule is relying on physicochemical characteristics of the tested compounds, including: (i) clogP ≤ 5; (ii) molecular weight (MW) ≤ 500 g/mol; (iii) number of hydrogen bond acceptors (HBA) (sum of N and O atoms) ≤ 10, (iv) number of hydrogen bond donors (HBD) (sum of OH and NH groups) ≤ 5. Other related criteria were added later [40]: (v) number of rotatable bonds (*n* Rotb) ≤ 10, (vi) total polar surface area (TPSA) ≤ 140 Å. We should remember that Lipinski’s rule of five offers a valuable tool to assess the drug likeliness, but it is mainly applicable for an orally administered drug. Based on the binding affinity scores of 153 molecules, the top three molecules with each receptor were chosen, resulting in the top four drug candidates. Further, drug likeness properties of these four phytocompounds were evaluated to predict the possible pharmacokinetics and pharmacodynamics of the compound (Table 1). The log P (logarithm of compound’s partition coefficient between *n*-octanol and water) value of the molecule is one of the major determining factors of the compound’s absorption, distribution in the body, penetration across vital membranes and biological barriers, metabolism, and excretion. Apigetrin is expected to have maximum lipophilicity as its log P value is 2.46, whereas xyloglucan, with a log P value of −1.74, is predicted to have the highest hydrophilicity. It implies that apigetrin will have poor aqueous solubility and dissolution, while permeation through biomembranes will be the limiting factor for xyloglucan. The sum of surfaces of polar atoms such as hydrogen, nitrogen, and oxygen in a molecule is calculated through TPSA, which is crucial for drug absorption through the human intestinal layer and drug penetration across the blood-brain barrier. TPSA should be in the range of 0–140 Å for a compound to be orally bioavailable. The TPSA value of apigetrin and curcumin is within the range and it clearly depicts that the hydrogen-bonding capacity of apigetrin and curcumin will be maximum, it is likely to have better bioavailability and membrane transport properties. A number of rotatable bonds indicated the more flexible nature of xyloglucan as compared to other compounds. A small molecule with a large surface area interacts well with water, enabling better drug absorption. All four compounds had molecular weights of less than 500 Da, implying that they could be easily transported, diffused across the cell membrane, and absorbed. Out of four compounds, apigetrin and curcumin fulfilled Lipinski’s rule of five criteria whereas cynaroside and xyloglucan violated the two rules of Lipinski’s. Interestingly, it has been noted that 16% of oral medications available in the market shows one violation and 6% fail to meet the two or more conditions [41].

The ProTox II web server was used to predict the LD_50_ value, toxicity class, and toxicity parameters. All the best identified compounds were predicted using various toxicity models, including the hepatotoxicity, carcinogenicity, immunotoxicity, mutagenicity, and cytotoxicity models. Out of four compounds, apigetrin, cynaroside and xyloglucan showed the highest LD_50_ values of 5000 mg/kg. These three compounds belong to the toxicity class 5. This toxicity class suggests that the compounds ‘might be harmful, if swallowed’. curcumin reported the lowest LD_50_ value of 2000 mg/kg with toxicity class 4 (‘harmful if swallowed’). Table 2 shows compounds with LD_50_ value with its toxicity class. Table 2 also summarise the effects of compounds on hepatotoxicity, carcinogenicity, immunotoxicity, mutagenicity, and cytotoxicity models. All four compounds were found to be inactive in terms of hepatotoxicity, carcinogenicity, and cytotoxicity. In contrast to apigetrin, which appeared to be mutagenic, the other three compounds have no effect on mutagenicity. Similarly, among the four compounds, only curcumin exhibits an immunotoxic nature.

Even though apigetrin and curcumin fulfilled the oral bioavailability criteria, the former was found to be mutagenic, while the latter was found to be positive for immunotoxicity. On the other hand, cynaroside and xyloglucan did not satisfy the rule of five criteria but appears to be non-toxic. Thus, a slight modification in their structure could aid in improving its poor bioavailability and toxic nature.

### 2.6. Bioactivity Score

**Figure 13:**
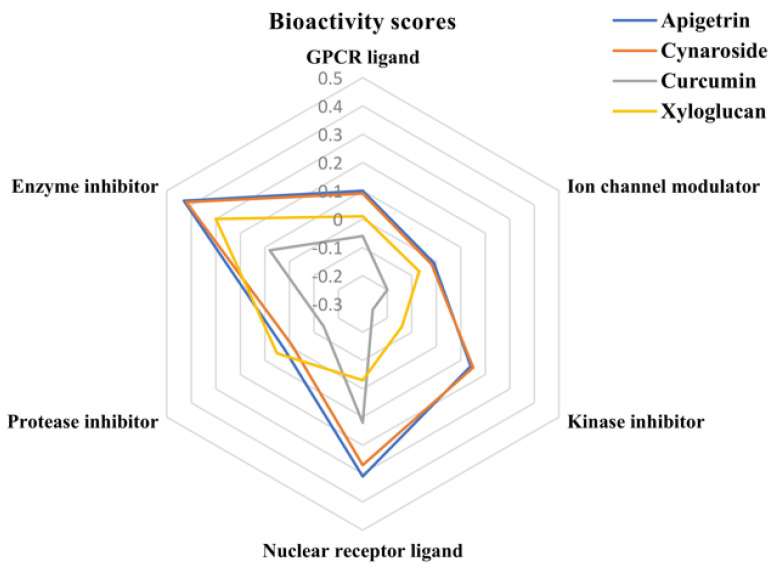
Bioactivity scores of apigetrin, cynaroside, curcumin and xyloglucan.

Table 3 summarizes the bioactivity score of the prime phytocompounds. A higher bioactivity score represents greater activity. Compounds with a bioactivity score more than 0.00 are supposed to have a significant effect, compounds with a value between −0.50 and 0.00 are presumed to have a moderate effect, and compounds with a score less than −0.50 are assumed to be inactive [42]. The bioactivity scores for the GPCR ligand (0.10), nuclear receptor ligand (0.27) and enzyme inhibitor (0.43) were found to be maximum for apigetrin, representing its strong interaction with the GPCR and Nuclear receptor ligands and indicates its potential as a strong enzyme inhibitor. The bioactivity score of cynaroside also demonstrates its strong interaction affinity for the GPCR ligand (0.09), nuclease receptor ligand (0.27) and significant enzyme inhibition activity (0.42). The bioactivity score for the protease inhibitor activity was maximum for xyloglucan i.e., 0.05. Curcumin and xyloglucan also exhibit a decisive enzyme inhibitory action with bioactivity scores of 0.08 and 0.30, respectively. It is worth mentioning that none of the phytocompounds scores less than −0.50 in any bioactivity segments (Figure 13). The results of the present study demonstrated that the investigated compounds are biologically active molecules and will produce the physiological actions by interacting with GPCR ligands, nuclear receptor ligands, and inhibit protease and other enzyme.

## 3. Materials and Methods

### 3.1. Protein Retrieval

The X-ray crystal structures of CDK8 with a resolution of 2.45 Å (PDB ID-6T41) [43], PR with a resolution of 2.41 Å (PDB ID-4OAR) [44] and EGFR with a resolution of 3.10 Å (PDB ID- 2J6M) [45] were obtained from the RCSB Protein Data Bank (https://www.rcsb.org/ accessed on 18 April 2022). Table 4 lists the proteins involved in the study and their descriptions.

### 3.2. Ligand Preparation

The five selected Indian medicinal plants (*Zingiber officinale* Roscoe, *Cuminum cyminum* L., *Piper nigrum* L., *Curcuma longa* L., and *Allium sativum* L.) contain 153 phytocompounds (Supplementary File Table S1). The structures of the phytocompounds, also termed as ligands were obtained from Pubchem database (https://pubchem.ncbi.nlm.nih.gov/ accessed on 22 April 2022) as a sdf file and converted into Mol, PDBQT and PDB file formats using OPEN BABEL software (https://openbabel.org/wiki/Main_Page/ accessed on 25 April 2022).

### 3.3. Molecular Docking

The obtained crystal structures of target proteins were prepared individually using chimera 1.15 software [46]. The following steps were considered: removal of water molecules and bound ions, addition of missing hydrogen atoms, and allocation of Kollman charges. The residues which were considered critical in binding of co-crystalized ligands were identified using pymol software [47]. It was assumed that binding location of the co-crystalized ligand is the protein’s active site and the amino acid residues which are crucial for binding of the co-crystallized ligand are active site residues (Table 2). The iGEMDOCK 2.1 [48] application was used to screen 153 compounds with each target protein for protein ligand interactions. The genetic algorithm parameters, which guided the docking procedure, were set as 200 (population size), 60 (generations), and 2 (number of solutions). The conformation with the lowest total binding energy among the different conformations generated was considered the best binding conformation of phytocompounds against target proteins. The identified phytocompounds were imported into the iGEMDOCK graphical user interface and were sorted by the post-docking analysis based on their binding energies and compound fitness score measured by the iGEMDOCK docking algorithm [35]. The top three compounds against each target protein were identified and subjected to further docking analysis using Autodock 4.2 [49]. Autodock 4.2 was used to optimise proteins by removing water and other atoms, and then by adding polar hydrogen groups. The graphical user interfaces that are associated with this software are MGL tools, Autodock tools, and Rasmol. For binding to take place, a grid box was set up surrounding the binding sites of the receptors. The grid box parameters for each receptor are mentioned in Table 5. The grid file was saved as a .gpf file and run through autogrid. The docking calculation was then carried out using the Lamarckian genetic algorithm with the predefined parameters (number of runs 10; population size 150; maximum number of generations 27,000). The dock file was saved as .dpf and after running autodock, the final results were obtained as .dlg file. The .dlg file contained all the information such as binding energy, binding residues etc. Interacting protein residues forming hydrogen bonds with phytocompounds were visualized using ligplot^+^ [50].

### 3.4. Drug Likeness and Toxicity Prediction

Drug likeness properties of the ligands were evaluated using Molinspiration cheminformatics software version 2011.06 [51]. Molinspiration offers a broad range of cheminformatics tools that are important for calculating critical molecular properties (Log P, polar surface area, number of hydrogen bond donors and acceptors and others) [52]. This platform also allows for evaluation of whether a given molecule violated any of Lipinski’s rule of five [39]. Molecules that do not violate the rule can be considered to have succeeded in pharmacokinetics tests, such as oral bioavailability. According to Google Scholar, molecular inspiration tools are cited more than 4500 times (www.molinspiration.com/ accessed on 21 June 2022). In our study drug-likeness properties including topological polar surface area (TPSA), octanol/water partition coefficient (Log P), number of hydrogen bond donors (NH and OH groups) and acceptors (mostly N and O groups), number of atoms, molecular weight, and number of rotatable bonds were calculated to justify the oral application of the compounds [53]. At the same time, the Protox II webserver (https://tox-new.charite.de/protox_II/ accessed on 21 June 2022) was used to predict the toxicity of the ligands [54]. ProTox-II predicts values of various toxicity endpoints such as hepatotoxicity, carcinogenicity, cytotoxicity, mutagenicity, immunotoxicity, along with a predicted median lethal dose (LD_50_) and toxicity class [54,55].

### 3.5. Bioactivity Score

A drug is supposed to bind with a biological target. Biological targets can be enzymes, ion channels, and receptors. Bioactivity of the phytocompounds can be checked by calculating the activity score of the GPCR ligand, ion channel modulator, nuclear receptor ligand, kinase inhibitor, protease inhibitor, enzyme inhibitor. All the parameters were checked with the help of molinspiration chemoinformatics software [51]. In the Molinspiration tool, the miscreen engine first analyses a training set of active structures (in extreme cases even a single active molecule is sufficient to build a usable model) and compares it with inactive molecules by using sophisticated Bayesian statistics. Only SMILES or SDF structures of active phytocompounds are sufficient for the training, no information about the active site or binding mode is necessary [56]. For organic molecules the probability is if the bioactivity score is (>0), then it is active, if (−5.0–0.0) then moderately active, if (<−5.0) then inactive [42].

## 4. Conclusions

Drugs derived from medicinal plants have traditionally been used to treat various diseases. Considering the carnage that breast cancer has caused for decades, finding an effective therapy is critical and urgent. Spice yielding plants are rich in phytocompounds, might be an effective tool to combat breast cancer. Our findings show that apigetrin, cynaroside, and curcumin have higher binding affinity in the active sites of PR, EGFR and CDK8 proteins and, most of the time, outperformed the standard drugs that are available in the market. ADME/T analysis revealed that apigetrin and curcumin fulfilled all the criteria to be orally bioavailable, but they were found to be positive for mutagenicity and immunotoxicity, respectively. The other compounds, cynaroside and xyloglucan, do not satisfy the rule of five criteria but appear non-toxic. Hence, these compounds demand structural optimization such as modifying the structure, changing an ionizable group, optimizing lipophilicity, isosteric substitution of polar groups, etc., to overcome the deficiencies or shortcomings by improving their pharmacokinetic or physicochemical parameters without compromising their efficacy. In the future, the identified phytocompounds can be validated through molecular dynamics simulations on the protein model and experimental research on animal models to confirm their status as novel compounds against breast cancer and pave the way for developing strongly targeted therapies against breast cancer.

## Figures and Tables

**Table 1:** Drug likeness score of compounds.

Sl No.	Compound Name	LogP (≤5)	TPSA (≤140 Å)	*n* Atoms	MW (≤500 da)	*n* ON (≤10)	*n* OHNH (≤5)	*n* Rotb	Volume	Violation of Rules
1.	Apigetrin	2.46	90.89	20	270.24	5	3	1	224.05	0
2.	Cynaroside	0.19	190.2	32	448.3	11	7	4	364.1	2
3.	Curcumin	2.30	93.07	27	368.38	6	2	8	332.18	0
4.	Xyloglucan	−1.74	212.67	34	488.44	13	7	12	415.44	2

**Table 2:** Toxicity prediction of the compounds.

Sl No.	Compound Name	LD_50_ (mg/kg)	Toxicity Class	Hepatotoxicity	Carcinogenicity	Immunotoxicity	Mutagenicity	Cytotoxicity
1.	Apigetrin	5000	5	N (0.82)	N (0.86)	N (0.93)	Y (0.59)	N (0.69)
2.	Cynaroside	5000	5	N (0.82)	N (0.85)	N (0.74)	N (0.76)	N (0.69)
3.	Curcumin	2000	4	N (0.61)	N (0.84)	Y (0.92)	N (0.88)	N (0.88)
4.	Xyloglucan	5000	5	N (0.86)	N (0.80)	N (0.99)	N (0.77)	N (0.80)

Probability: Y (Yes, Active), N (No, Inactive).

**Table 3:** Bioactivity score of the compounds according to Molinspiration Cheminformatics software.

Sl No.	Compound Name	GPCR Ligand	Ion Channel Modulator	Kinase Inhibitor	Nuclear Receptor Ligand	Protease Inhibitor	Enzyme Inhibitor
**1.**	Apigetrin	0.10	−0.01	0.14	0.31	0.02	0.43
**2.**	Cynaroside	0.09	−0.02	0.15	0.27	−0.01	0.42
**3.**	Curcumin	−0.06	−0.20	−0.26	0.12	−0.14	0.08
**4.**	Xyloglucan	0.01	−0.07	−0.14	−0.03	0.05	0.30

**Table 4:** Target proteins and their functions.

Protein	Full Name of the Protein	Protein Function	PDB ID
CDK8	Cyclin-dependent kinase 8	The Notch intracellular domain, SREBP (Sterol regulatory-element binding proteins), and STAT1-S727 are all phosphorylated by CDK8. By modulating the turnover of subunits in the mediator complex tail module, CDK8 also represses transcriptional activity. CDK8 also affects interaction of RNA polymerase II with the mediator complex.	6T41
PR	Progesterone receptor	Proliferation and differentiation of cell, transcriptional activator and repressor.	4OAR
EGFR	Epidermal growth factor receptor	When a ligand binds to an epidermal growth factor receptor, the receptor is able to attach (dimerize) with another epidermal growth factor receptor protein nearby, turning on (activating) the receptor complex. As a result, cell signalling cascades that promote cell growth and division (proliferation) as well as cell survival are activated.	2J6M

**Table 5:** Active site residues and grid box parameters selected for target proteins.

Target Proteins	Active Site Residues	Grid Box Parameters	
		x Centre × y Centre × z Centre	Number of Points in x-Dimension × Number of Points in y-Dimension × Number of Points in z-Dimension
CDK8	ILE-79, TYR-99, LEU-158, ALA-100	−5.797 × −8.769 × 12.8	60 × 60 × 60
PR	GLY-722, GLN-725, ARG-766, THR-894	12.542 × 27.621 × 15.492	60 × 60 × 60
EGFR	THR-854, THR-790, GLN-791, LEU-792, MET-793	−56.344 × −0.481 × −24.16	60 × 60 × 60

## Data Availability

All data are contained within the article.

## Supplementary Materials

The following supporting information can be downloaded at: https://www.mdpi.com/article/10.3390/molecules27196590/s1, Table S1: List of phytocompounds from five common Indian spices; Table S2: Phytocompounds from five plants that overlapped; Table S3: Binding energy scores calculated by iGEMDOCK 2.1 for 153 phytocompounds against CDK8, PR and EGFR target proteins.

## References

[1] Fouad Y.A., Aanei C. (2017). Revisiting the hallmarks of cancer. Am. J. Cancer Res..

[2] Sung H., Ferlay J., Siegel R.L., Laversanne M., Soerjomataram I., Jemal A., Bray F. (2021). Global cancer statistics 2020: GLOBOCAN estimates of incidence and mortality worldwide for 36 cancers in 185 countries. CA Cancer J. Clin..

[3] WHO Breast Cancer. https://www.who.int/news-room/fact-sheets/detail/breast-cancer.

[4] Sahayarayan J.J., Rajan K.S., Vidhyavathi R., Nachiappan M., Prabhu D., Alfarraj S., Arokiyaraj S., Daniel A.N. (2021). In-silico protein-ligand docking studies against the estrogen protein of breast cancer using pharmacophore based virtual screening approaches. Saudi J. Biol. Sci..

[5] Ataollahi M.R., Sharifi J., Paknahad M.R., Paknahad A. (2015). Breast cancer and associated factors: A review. J. Med. Life.

[6] Kim B., Fatayer H., Hanby A.M., Horgan K., Perry S.L., Valleley E.M., Verghese E.T., Williams B.J., Thorne J.L., Hughes T.A. (2013). Neoadjuvant chemotherapy induces expression levels of breast cancer resistance protein that predict disease-free survival in breast cancer. PLoS ONE.

[7] Greenwell M., Rahman P.K.S.M. (2015). Medicinal plants: Their use in anticancer treatment. Int. J. Pharm. Sci. Rev. Res..

[8] Iqbal J., Abbasi B.A., Mahmood T., Kanwal S., Ali B., Shah S.A., Khalil A.T. (2017). Plant-derived anticancer agents: A green anticancer approach. Asian Pac. J. Trop. Biomed..

[9] Ekins S., Mestres J., Testa B. (2007). In silico pharmacology for drug discovery: Methods for virtual ligand screening and profiling. Br. J. Pharmacol..

[10] Mullard A. (2014). New drugs cost US$2.6 billion to develop. Nat. Rev. Drug. Discov..

[11] Li K., Du Y., Li L., Wei D.Q. (2020). Bioinformatics approaches for anti-cancer drug discovery. Curr. Drug Targets.

[12] Wang G., Zhu W. (2016). Molecular docking for drug discovery and development: A widely used approach but far from perfect. Future Med. Chem..

[13] Blundell T.L., Jhoti H., Abell C. (2002). High-throughput crystallography for lead discovery in drug design. Nat. Rev. Drug. Discov..

[14] Bajorath J. (2002). Integration of virtual and high-throughput screening. Nat. Rev. Drug. Discov..

[15] Issa N.T., Badiavas E.V., Schürer S. (2019). Research techniques made simple: Molecular docking in dermatology-A foray into in silico drug discovery. J. Investig. Dermatol..

[16] Gohlke H., Klebe G. (2002). Approaches to the description and prediction of the binding affinity of small-molecule ligands to macromolecular receptors. Angew. Chem. Int. Ed..

[17] Kuntz I.D., Blaney J.M., Oatley S.J., Langridge R., Ferrin T.E. (1982). A geometric approach to macromolecule-ligand interactions. J. Mol. Biol..

[18] Bartuzi D., Kaczor A.A., Targowska-Duda K.M., Matosiuk D. (2017). Recent advances and applications of molecular docking to G protein-coupled receptors. Molecules.

[19] DesJarlais R.L., Dixon J.S. (1994). A shape-and chemistry-based docking method and its use in the design of HIV-1 protease inhibitors. J. Comput. Aided Mol..

[20] Venhorst J., ter Laak A.M., Commandeur J.N., Funae Y., Hiroi T., Vermeulen N.P. (2003). Homology modeling of rat and human cytochrome P450 2D (CYP2D) isoforms and computational rationalization of experimental ligand-binding specificities. J. Med. Chem..

[21] Williams P.A., Cosme J., Ward A., Angove H.C., Matak Vinković D., Jhoti H. (2003). Crystal structure of human cytochrome P450 2C9 with bound warfarin. Nature.

[22] Padma V.V. (2015). An overview of targeted cancer therapy. BioMedicine.

[23] Acharya R., Chacko S., Bose P., Lapenna A., Pattanayak S.P. (2019). Structure based multitargeted molecular docking analysis of selected furanocoumarins against breast cancer. Sci. Rep..

[24] Jha V., Devkar S., Gharat K., Kasbe S., Matharoo D.K., Pendse S., Bhosale A., Bhargava A. (2022). Screening of Phytochemicals as Potential Inhibitors of Breast Cancer using Structure Based Multitargeted Molecular Docking Analysis. Phytomed. Plus..

[25] Daniel A.R., Hagan C.R., Lange C.A. (2011). Progesterone receptor action: Defining a role in breast cancer. Expert Rev. Endocrinol. Metab..

[26] Masuda H., Zhang D., Bartholomeusz C., Doihara H., Hortobagyi G.N., Ueno N.T. (2012). Role of epidermal growth factor receptor in breast cancer. Breast Cancer Res. Treat..

[27] Galbraith M.D., Donner A.J., Espinosa J.M. (2010). CDK8: A positive regulator of transcription. Transcription.

[28] Crown J. (2017). CDK8: A new breast cancer target. Oncotarget.

[29] Rajagopal K., Byran G., Jupudi S., Vadivelan R. (2020). Activity of phytochemical constituents of black pepper, ginger, and garlic against coronavirus (COVID-19): An in silico approach. Int. J. Health Allied Sci..

[30] Aftab A., Khan R., Shah W., Azhar M., Unar A., Jafar Hussain H.M., Waqas A. (2021). Computational analysis of Cyclin D1 gene SNPs and association with breast cancer. Biosci. Rep..

[31] Meng X.Y., Zhang H.X., Mezei M., Cui M. (2011). Molecular docking: A powerful approach for structure-based drug discovery. Curr. Comput.-Aided Drug Des..

[32] Siegel R.L., Miller K.D., Jemal A. (2019). Cancer statistics. Cancer J. Clin..

[33] Ashfaq U.A., Mumtaz A., ul Qamar T., Fatima T. (2013). MAPS database: Medicinal plant activities, phytochemical and structural database. Bioinformation.

[34] Gagic Z., Ruzic D., Djokovic N., Djikic T., Nikolic K. (2020). In silico methods for design of kinase inhibitors as anticancer drugs. Front. Chem..

[35] Iheagwam F.N., Ogunlana O.O., Ogunlana O.E., Isewon I., Oyelade J. (2019). Potential anti-cancer flavonoids isolated from Caesalpinia bonduc young twigs and leaves: Molecular docking and in silico studies. Bioinform. Biol. Insights.

[36] Kim S.M., Vetrivel P., Ha S.E., Kim H.H., Kim J.A., Kim G.S. (2020). Apigetrin induces extrinsic apoptosis, autophagy and G2/M phase cell cycle arrest through PI3K/AKT/mTOR pathway in AGS human gastric cancer cell. J. Nutr. Biochem..

[37] Ji J., Wang Z., Sun W., Li Z., Cai H., Zhao E., Cui H. (2021). Effects of Cynaroside on Cell Proliferation, Apoptosis, Migration and Invasion though the MET/AKT/mTOR Axis in Gastric Cancer. Int. J. Mol. Sci..

[38] Liu H.T., Ho Y.S. (2018). Anticancer effect of curcumin on breast cancer and stem cells. Food Sci. Hum. Wellness.

[39] Lombardo F., Lipinski C.A., Dominy B.W., Feeney P.J. (1997). Experimental and Computational Approaches to Estimate Solubility and Permeability in Drug Discovery and Development Setting. Adv. Drug Deliv. Rev..

[40] Veber D.F., Johnson S.R., Cheng H.Y., Smith B.R., Ward K.W., Kopple K.D. (2002). Molecular properties that influence the oral bioavailability of drug candidates. J. Med. Chem..

[41] Bickerton G.R., Paolini G.V., Besnard J., Muresan S., Hopkins A.L. (2012). Quantifying the chemical beauty of drugs. Nat. Chem..

[42] Verma A. (2012). Lead finding from *Phyllanthus debelis* with hepatoprotective potentials. Asian Pac. J. Trop. Biomed..

[43] Klatt F., Leitner A., Kim I.V., Ho-Xuan H., Schneider E.V., Langhammer F., Weinmann R., Müller M.R., Huber R., Meister G. (2020). A precisely positioned MED12 activation helix stimulates CDK8 kinase activity. Proc. Natl. Acad. Sci. USA.

[44] Petit-Topin I., Fay M., Resche-Rigon M., Ulmann A., Gainer E., Rafestin-Oblin M.E., Fagart J. (2014). Molecular determinants of the recognition of ulipristal acetate by oxo-steroid receptors. J. Steroid Biochem. Mol. Biol..

[45] Yun C.H., Boggon T.J., Li Y., Woo M.S., Greulich H., Meyerson M., Eck M.J. (2007). Structures of lung cancer-derived EGFR mutants and inhibitor complexes: Mechanism of activation and insights into differential inhibitor sensitivity. Cancer Cell.

[46] Pettersen E.F., Goddard T.D., Huang C.C., Couch G.S., Greenblatt D.M., Meng E.C., Ferrin T.E. (2004). UCSF Chimera—A visualization system for exploratory research and analysis. J. Comput. Chem..

[47] Schrodinger L., DeLano W. (2020). PyMol. http://www.pymol.org/pymol.

[48] Hsu K.C., Chen Y.F., Lin S.R., Yang J.M. (2011). iGEMDOCK: A graphical environment of enhancing GEMDOCK using pharmacological interactions and post-screening analysis. BMC Bioinform..

[49] Morris G.M., Huey R., Lindstrom W., Sanner M.F., Belew R.K., Goodsell D.S., Olson A.J. (2009). AutoDock4 and AutoDockTools4: Automated docking with selective receptor flexibility. J. Comput. Chem..

[50] Laskowski R.A., Swindells M.B. (2011). LigPlot+: Multiple ligand-protein interaction diagrams for drug discovery. J. Chem. Inf. Model..

[51] (2021). Molinspiration Cheminformatics free web services. https://www.molinspiration.com.

[52] Fernandes G.S., de Moura Pereira M.B., Marinho A.C.B., Machado B., Moreira A.C., de Freitas M.P., Lang K.L., Antunes J.E. (2015). In Silico Pharmacokinetics Studies for Quinazolines Proposed as EGFR Inhibitors. Open J. Med. Chem..

[53] Srivastava R. (2021). Theoretical Studies on the Molecular Properties, Toxicity, and Biological Efficacy of 21 New Chemical Entities. ACS Omega.

[54] Ghosh S., Tripathi P., Talukdar P., Talapatra S.N. (2019). In silico study by using ProTox-II webserver for oral acute toxicity, organ toxicity, immunotoxicity, genetic toxicity endpoints, nuclear receptor signalling and stress response pathways of synthetic pyrethroids. World Sci. News.

[55] Bhat V., Chatterjee J. (2021). The use of in silico tools for the toxicity prediction of potential inhibitors of SARS-CoV-2. Altern. Lab. Anim..

[56] Mishra S.S., Kumar N., Singh H.P., Ranjan S., Sharma C.S. (2018). In silico pharmacokinetic, bioactivity and toxicity study of some selected anti-asthmatic agents. Int. J. Pharm. Sci. Drug. Res..

