# Supplementary material for: Natural Phytocompounds from Common Indian Spices for Identification of Three Potential Inhibitors of Breast Cancer: A Molecular Modelling Approach
Source: Molecules. 2022 Oct 5;27(19):6590. doi: 10.3390/molecules27196590 (PMC9573590; doi:10.3390/molecules27196590)
Supplement: Supplementary file 1 [file molecules-27-06590-s001.zip › molecules-1876501-supplementary.pdf]

Table S1: List of phytocompounds from five common Indian spices

Sl.No	Pubchem ID	Name of the phytocompounds	Canonical SMILES
**1.** ***Zingiber officinale*** **Roscoe**			
1	44559528	(S)-6-Gingerol	CCCCCC(CC(=O)CCC1=CC(=C(C=C1)OC)O)O
2	92139	Alpha-curcumene	CC1=CC=C(C=C1)C(C)CCC=C(C)C
3	7140311	(+)-Angelicoidenol	CC1(C2CC(C1(CC2O)C)O)C
4	442484	Beta phellandrene	CC(C)C1CCC(=C)C=C1
5	6552009	(+)-Borneol	CC1(C2CCC1(C(C2)O)C)C
6	2758	1,8-Cineole	CC1(C2CCC(O1)(CC2)C)C
7	5318274	[4]-Gingerdiol 3,5-diacetate	CCCC(CC(CCC1=CC(=C(C=C1)O)OC)OC(=O)C)OC(=O)C
8	10987383	10-Epizonarene	CC1CCC(=C2C1CCC(=C2)C)C(C)C
9	5317591	10-Gingerdione	CCCCCCCCCC(=O)C=C(CCC1=CC(=C(C=C1)O)OC)O
10	442793	6-Gingerol	CCCCCC(CC(=O)CCC1=CC(=C(C=C1)O)OC)O
11	6442612	10-shogaol	CCCCCCCCCC=CC(=O)CCC1=CC(=C(C=C1)O)OC
12	86196540	12-Gingediol	CCCCCCCCCCCC(CC(CCC1=CC(=C(C=C1)O)OC)O)O
13	5317599	12-Gingerol	CCCCCCCCCCCC(CC(=O)CCC1=CC(=C(C=C1)O)OC)O
14	5317596	4-Gingerol	CCCC(CC(=O)CCC1=CC(=C(C=C1)O)OC)O
15	11127403	Alpha-Zingiberene	CC1=CCC(C=C1)C(C)CCC=C(C)C
16	7462	Alpha-Terpinene	CC1=CC=C(CC1)C(C)C
17	17100	Alpha-Terpineol	CC1=CCC(CC1)C(C)(C)O
18	65575	Alpha-Cedrol	CC1CCC2C13CCC(C(C3)C2(C)C)(C)O
19	19725	Alpha-Copaene	CC1=CCC2C3C1C2(CCC3C(C)C)C
20	442359	Alpha-Cubebene	CC1CCC(C2C13C2C(=CC3)C)C(C)C
21	5281516	Alpha-Farnesene	CC(=CCCC(=CCC=C(C)C=C)C)C
22	5280934	Linolenic acid	CCC=CCC=CCC=CCCCCCCCC(=O)O
23	7460	Alpha Phellandrene	CC1=CCC(C=C1)C(C)C
24	6654	Alpha-Pinene	CC1=CCC2CC1C2(C)C
25	10856614	Alpha Selinene	CC1=CCCC2(C1CC(CC2)C(=C)C)C
26	853433	Isoeugenol	CC=CC1=CC(=C(C=C1)O)OC
27	6918391	Beta-Elemene	CC(=C)C1CCC(C(C1)C(=C)C)(C)C=C
28	222284	Beta-sitosterol	CCC(CCC(C)C1CCC2C1(CCC3C2CC=C4C3(CCC(C4)O)C)C)C(C)C
29	5280863	Kaempferol	C1=CC(=CC=C1C2=C(C(=O)C3=C(C=C(C=C3O2)O)O)O)O
30	969516	Curcumin	COC1=C(C=CC(=C1)C=CC(=O)CC(=O)C=CC2=CC(=C(C=C2)O)OC)O
**2.** ***Cuminum cyminum*** **L.**			
31	2758	1,8-Cineole	CC1(C2CCC(O1)(CC2)C)C
32	7460	Alpha phellandrene	CC1=CCC(C=C1)C(C)C
33	6654	Alpha Pinene	CC1=CCC2CC1C2(C)C
34	5280704	Apigetrin	C1=CC(=CC=C1C2=CC(=O)C3=C(C=C(C=C3O2)OC4C(C(C(C(O4)CO)O)O)O)O)O
35	6918391	Beta-Elemene	CC(=C)C1CCC(C(C1)C(=C)C)(C)C=C
36	222284	Beta sitosterol	CCC(CCC(C)C1CCC2C1(CCC3C2CC=C4C3(CCC(C4)O)C)C)C(C)C
37	10856614	Alpha Selinene	CC1=CCCC2(C1CC(CC2)C(=C)C)C
38	5280443	Apigenin	C1=CC(=CC=C1C2=CC(=O)C3=C(C=C(C=C3O2)O)O)O

39	5273469	Benzyl cinnamate	C1=CC=C(C=C1)COC(=O)C=CC2=CC=CC=C2
40	10104370	Beta-bisabolene	CC1=CCC(CC1)C(=C)CCC=C(C)C
41	5280489	Beta Carotene	CC1=C(C(CCC1)(C)C)C=CC(=CC=CC (=CC=CC=C(C)C=CC=C(C)C=CC2=C(CCCC2(C)C)C)C)C
42	17868	Alpha-Thujene	CC1=CCC2(C1C2)C(C)C
43	5281517	Beta-farnesene	CC(=CCCC(=CCCC(=C)C=C)C)C
44	442484	Beta phellandrene	CC(C)C1CCC(=C)C=C1
45	14896	Beta-pinene	CC1(C2CCC(=C)C1C2)C
46	31244	Anisaldehyde	COC1=CC=C(C=C1)C=O
47	66841	Beta-terpinene	CC(C)C1=CCC(=C)CC1
48	6448	Bornyl acetate	CC(=O)OC1CC2CCC1(C2(C)C)C
49	173183	Campesterol	CC(C)C(C)CCC(C)C1CCC2C1(CCC3C2CC=C4C3(CCC(C4)O)C)C
50	6616	Camphene	CC1(C2CCC(C2)C1=C)C
51	7438	Carveol	CC1=CCC(CC1O)C(=C)C
52	1742210	Caryophyllene oxide	CC1(CC2C1CCC3(C(O3)CCC2=C)C)C
53	5281515	Caryophyllene	CC1=CCCC(=C)C2CC(C2CC1)(C)C
54	305	Choline	C[N+](C)(C)CCO
55	326	Cuminaldehyde	CC(C)C1=CC=C(C=C1)C=O
56	10820	Cuminic acid	CC(C)C1=CC=C(C=C1)C(=O)O
57	5280637	Cynaroside	C1=CC(=C(C=C1C2=CC(=O)C3=C(C=C(C=C3O2)OC4C(C(C(C(O4)CO)O)O)O)O)O)O
58	3314	Eugenol	COC1=C(C=CC(=C1)CC=C)O
59	445070	Farnesol	CC(=CCCC(=CCCC(=CCO)C)C)C
60	5281522	Isocaryophyllene	CC1=CCCC(=C)C2CC(C2CC1)(C)C
61	22311	Limonene	CC1=CCC(CC1)C(=C)C
62	6549	Linalool	CC(=CCCC(C)(C=C)O)C
63	5280445	Luteolin	C1=CC(=C(C=C1C2=CC(=O)C3=C(C=C(C=C3O2)O)O)O)O
64	31253	Myrcene	CC(=CCCC(=C)C=C)C
65	10582	Myrtenol	CC1(C2CC=C(C1C2)CO)C
66	938	Niacin	C1=CC(=CN=C1)C(=O)O
67	445639	Oleic acid	CCCCCCCCC=CCCCCCCCC(=O)O
68	7463	P-cymene	CC1=CC=C(C=C1)C(C)C
69	10247670	Piperitol	COC1=C(C=CC(=C1)C2C3COC(C3CO2)C4=CC5=C(C=C4)OCO5)O
70	6987	Piperitone	CC1=CC(=O)C(CC1)C(C)C
71	6989	Thymol	CC1=CC(=C(C=C1)C(C)C)O
**3. *Piper nigrum* L.**			
72	22311	Limonene	CC1=CCC(CC1)C(=C)C
73	117443	Cubebin	C1C(C(C(O1)O)CC2=CC3=C(C=C2)OCO3)CC4=CC5=C(C=C4)OCO5
74	19725	Alpha-Copaene	CC1=CCC2C3C1C2(CCC3C(C)C)C

75	5280450	Linoleic acid	CCCCCC=CCC=CCCCCCCCC(=O)O
76	32735	Beta-pinone	CC1(C2CCC(=O)C1C2)C
77	10467	Arachidic acid	CCCCCCCCCCCCCCCCCCCC(=O)O
78	54670067	Ascorbic acid	C(C(C1C(=C(C(=O)O1)O)O)O)O
79	10104370	Beta-bisabolene	CC1=CCC(CC1)C(=C)CCC=C(C)C
80	5281515	Beta-caryophyllene	CC1=CCCC(=C)C2CC(C2CC1)(C)C
81	6918391	Beta elemene	CC(=C)C1CCC(C(C1)C(=C)C)(C)C=C
82	14985	Alpha tocopherol	CC1=C(C2=C(CCC(O2)(C)CCCC(C)CCCC(C)CCCC(C)C)C(=C1O)C)C
83	442393	Beta-selinene	CC(=C)C1CCC2(CCCC(=C)C2C1)C
84	222284	Beta sitosterol	CCC(CCC(C)C1CCC2C1(CCC3C2CC=C4C3(CCC(C4)O)C)C)C(C)C
85	14350	Epoxydihydrocaryophyllene	CC1(CC2C1CCC3(C(O3)CCC2=C)C)C
86	5280863	Kaempferol	C1=CC(=CC=C1C2=C(C(=O)C3=C(C=C(C=C3O2)O)O)O)O
87	65575	Alpha-Cedrol	CC1CCC2C13CCC(C(C3)C2(C)C)(C)O
88	638011	Citral	CC(=CCCC(=CC=O)C)C
89	7794	Citronellal	CC(CCC=C(C)C)CC=O
90	129700027	Citronellal acetate	CC(CCC=C(C)C)CC(=O)OC(=O)C
91	92780	Cryptone	CC(C)C1CCC(=O)C=C1
92	441005	Delta cadinene	CC1=CC2C(CCC(=C2CC1)C)C(C)C
93	518975	Calamene	CC1CCC2=C(C=CC(=C2C1)C(C)C)C
94	119	GABA	C(CC(=O)O)CN
95	1549026	Geranyl acetate	CC(=CCCC(=CCOC(=O)C)C)C
96	8438	Heliotropine	C1OC2=C(O1)C=C(C=C2)C=O
97	94741	Hentriacontan-16-one	CCCCCCCCCCCCCCC(=O)CCCCCCCCCCCCCCC
98	12410	Hentriacontane	CCCCCCCCCCCCCCCCCCCCCCCCCCCCCCC
99	68345	Hentriacontanol	CCCCCCCCCCCCCCCCCCCCCCCCCCCCCCCO
100	170833	Isopulegol	CC1CCC(C(C1)O)C(=C)C
101	173183	Campesterol	CC(C)C(C)CCC(C)C1CCC2C1(CCC3C2CC=C4C3(CCC(C4)O)C)C
102	527428	Limonene-4-ol	CC1=CCC(CC1)(C(=C)C)O
103	8294	Linalyl acetate	CC(=CCCC(C)(C=C)OC(=O)C)C
104	55255958	Methyl caffeic acid	CC(=CC1=CC(=C(C=C1)O)O)C(=O)O
105	637520	Methyl cinnamate	COC(=O)C=CC1=CC=CC=C1
106	80790	Methyl carvacrol	CC1=C(C=C(C=C1)C(C)C)OC
107	11493622	Monoterpenes	CC(=C(CCC(=CBr)C(=C)Cl)Br)C
108	4276	Myristicin	COC1=CC(=CC2=C1OCO2)CC=C
109	17429	N-formylpiperidine	C1CCN(CC1)C=O
110	5280343	Quercetin	C1=CC(=C(C=C1C2=C(C(=O)C3=C(C=C(C=C3O2)O)O)O)O)O
111	25202482	Sesquisabinene	CC(CCC=C(C)C)C12CCC(=C)C1C2
112	11230	Terpinen-4-OL	CC1=CCC(CC1)(C(C)C)O

**4. *Curcuma longa* L.**			
113	2758	1,8-Cineole	CC1(C2CCC(O1)(CC2)C)C
114	7460	Alpha phellandrene	CC1=CCC(C=C1)C(C)C
115	558221	AR-Tumerone	CC1=CC=C(C=C1)C(C)CC(=O)C=C(C)C
116	7462	Alpha terpinene	CC1=CC=C(CC1)C(C)C
117	17100	Alpha terpineol	CC1=CCC(CC1)C(C)(C)O
118	14985	Alpha tocopherol	CC1=C(C2=C(CCC(O2)(C)CCCC(C)CCCC(C)CCCC(C)C)C(=C1O)C)C
119	6654	Alpha pinene	CC1=CCC2CC1C2(C)C
120	222284	Beta sitosterol	CCC(CCC(C)C1CCC2C1(CCC3C2CC=C4C3(CCC(C4)O)C)C)C(C)C
121	444539	Cinnamic acid	C1=CC=C(C=C1)C=CC(=O)O
122	69879809	Cyclocurcumin	COC1=C(C=CC(=C1)C=CC2=CC(=O)CC(O2)C3=CC(=C(C=C3)O)OC)O
123	5469424	Demethoxycurcumin	COC1=C(C=CC(=C1)C=CC(=O)CC(=O)C=CC2=CC=C(C=C2)O)O
124	493570	Riboflavin	CC1=CC2=C(C=C1C)N(C3=NC(=O)NC(=O)C3=N2)CC(C(C(CO)O)O)O
125	3033866	Bisabolene	CC1=CCC(=C(C)CCC=C(C)C)CC1
126	5315469	Bisacumol	CC1=CC=C(C=C1)C(C)CC(C=C(C)C)O
127	14287397	Bisacurone	CC(CC(=O)C=C(C)C)C1CC(C(C=C1)(C)O)O
128	6552009	(+)-Borneol	CC1(C2CCC1(C(C2)O)C)C
129	689043	Caffeic acid	C1=CC(=C(C=C1C=CC(=O)O)O)O
130	637429	Calebin-A	COC1=C(C=CC(=C1)C=CC(=O)COC(=O)C=CC2=CC(=C(C=C2)O)OC)O
131	173183	Campesterol	CC(C)C(C)CCC(C)C1CCC2C1(CCC3C2CC=C4C3(CCC(C4)O)C)C
132	6616	Camphene	CC1(C2CCC(C2)C1=C)C
133	2537	Camphor	CC1(C2CCC1(C(=O)C2)C)C
134	379	Caprylic acid	CCCCCCCC(=O)O
135	26049	car-3-ene	CC1=CCC2C(C1)C2(C)C
136	3081930	Curzerenone	CC1=COC2=C1C(=O)C(C(C2)(C)C=C)C(=C)C
137	92139	Alpha Curcumene	CC1=CC=C(C=C1)C(C)CCC=C(C)C
138	167812	Curcumenol	CC1CCC2C13CC(=C(C)C)C(O3)(C=C2C)O
139	969516	Curcumin	COC1=C(C=CC(=C1)C=CC(=O)CC(=O)C=CC2=CC(=C(C=C2)O)OC)O
140	14240392	Curcumol	CC1CCC2C13CC(C(O3)(CC2=C)O)C(C)C
141	6441391	Curdione	CC1CCC=C(CC(=O)C(CC1=O)C(C)C)C
142	196216	Curlone	CC(CC(=O)C=C(C)C)C1CCC(=C)C=C1
143	5280489	Beta-carotene	CC1=C(C(CCC1)(C)C)C=CC(=CC=CC(=CC=CC=C(C)<br>C=CC=C(C)C=CC2=C(CCCC2(C)C)C)C)C
144	12315492	Beta-sesquiphellandrene	CC(CCC=C(C)C)C1CCC(=C)C=C1
145	10104370	Beta bisabolene	CC1=CCC(CC1)C(=C)CCC=C(C)C
146	3314	Eugenol	COC1=C(C=CC(=C1)CC=C)O
147	9548705	Germacrene	CC1=CCCC(=CCC(CC1)C(=C)C)C

148	460	Guaiacol	COC1=CC=CC=C1O
149	9548703	Guaiane	CC1CCC(CC2C1CCC2C)C(C)C
150	22311	Limonene	CC1=CCC(CC1)C(=C)C
151	6549	Linalool	CC(=CCCC(C)(C=C)O)C
152	938	Niacin	C1=CC(=CN=C1)C(=O)O
153	637542	P-Coumaric acid	C1=CC(=CC=C1C=CC(=O)O)O
154	8486	Vanillic acid	COC1=C(C=CC(=C1)C(=O)O)O
**5. *Allium sativum* L.**			
155	133337	2-vinyl-1,3-dithiine	C=CC1SCC=CS1
156	444899	Arachidonic acid	CCCCCC=CCC=CCC=CCC=CCCCC(=O)O
157	94204	24-methylenecycloartanol	CC(C)C(=C)CCC(C)C1CCC2(C1(CCC34C2CCC5C3(C4)CCC(C5(C)C)O)C)C
158	5386591	Ajoene	C=CCSSC=CCS(=O)CC=C
159	65036	Allicin	C=CCSS(=O)CC=C
160	87310	Alliin	C=CCS(=O)CC(C(=O)O)N
161	86374	Allixin	CCCCCC1=C(C(=O)C(=C(O1)C)OC)O
162	21612418	Allyl 2-propenethiosulfinate	C=CCOS(=S)CC=C
163	62434	Allyl methyl disulfide	CSSCC=C
164	129712276	Allyl methyl thiosulfinate	COS(=O)SCC=C
165	61926	Allyl methyl trisulfide	CSSSCC=C
166	5352855	Allylpropenyl disulfide	CC=CSSCC=C
167	7460	Alpha phellandrene	CC1=CCC(C=C1)C(C)C
168	5280443	Apigenin	C1=CC(=CC=C1C2=CC(=O)C3=C(C=C(C=C3O2)O)O)O
169	10219489	Vinyldithiin	C=CC1=CC=CSS1
170	5280863	Kaempferol	C1=CC(=CC=C1C2=C(C(=O)C3=C(C=C(C=C3O2)O)O)O)O
171	10717615	Beta chlorogenin	CC1CCC2(C(C3C(O2)CC4C3(CCC5C4CC(C6C5(CCC(C6)O)C)O)C)C)OC1
172	6444001	Xyloglucan	COC1=C(C=CC(=C1)C=CC(=O)OC2COC(C(C2O)O)OCC(C(C(C(C=O)O)O)O)O)O
173	6857447	Beta tocopherol	CC1=CC(=C(C2=C1OC(CC2)(C)CCCC(C)CCCC(C)CCCC(C)C)C)O
174	171548	Biotin	C1C2C(C(S1)CCCCC(=O)O)NC(=O)N2
175	689043	Caffeic acid	C1=CC(=C(C=C1C=CC(=O)O)O)O
176	1794427	Chlorogenic acid	C1C(C(C(CC1(C(=O)O)O)OC(=O)C=CC2=CC(=C(C=C2)O)O)O)O
177	638011	Citral	CC(=CCCC(=CC=O)C)C
178	9750	Citrulline	C(CC(C(=O)O)N)CNC(=O)N
179	16590	Diallyl disulfide	C=CCSSCC=C
180	866	Endolysin	C(CCN)CC(C(=O)O)N
181	445858	Ferulic acid	COC1=C(C=CC(=C1)C=CC(=O)O)O

182	637566	Geraniol	CC(=CCCC(=CCO)C)C
183	5281166	Jasmonic acid	CCC=CCC1C(CCC1=O)CC(=O)O
184	801	Auxin	C1=CC=C2C(=C1)C(=CN2)CC(=O)O
185	5280450	Linoleic acid	CCCCCC=CCC=CCCCCCCCC(=O)O
186	5280934	Linolenic acid	CCC=CCC=CCC=CCCCCCCCC(=O)O
187	11005	Myrisitic acid	CCCCCCCCCCCCCC(=O)O
188	938	Niacin	C1=CC(=CN=C1)C(=O)O
189	10494	Oleanolic acid	CC1(CCC2(CCC3(C(=CCC4C3(CCC5C4(CCC(C5(C)C)O)C)C)C2C1)C)C(=O)O)C
190	445639	Oleic acid	CCCCCCCCC=CCCCCCCCC(=O)O
191	6613	Pantothenic acid	CC(C)(CO)C(C(=O)NCCC(=O)O)O
192	441476	Pectin	C1(C(C(OC(C1O)O)C(=O)O)O)O
193	5280343	Quercetin	C1=CC(=C(C=C1C2=C(C(=O)C3=C(C=C(C=C3O2)O)O)O)O)O
194	338	Salicyclic acid	C1=CC=C(C(=C1)C(=O)O)O
195	54670067	Ascorbic acid	C(C(C1C(=C(C(=O)O1)O)O)O)O
196	637775	Sinapic acid	COC1=CC(=CC(=C1O)OC)C=CC(=O)O
197	5280794	Stigmasterol	CCC(C=CC(C)C1CCC2C1(CCC3C2CC=C4C3(CCC(C4)O)C)C)C(C)C
198	1110	Succinic acid	C(CC(=O)O)C(=O)O
199	5570	Trigonelline	C[N+]1=CC=CC(=C1)C(=O)[O-]
200	8486	Vanillic acid	COC1=C(C=CC(=C1)C(=O)O)O

***Green colour indicates the presence of molecules in more than one plant species**

Table S2: Phytocompounds from five plants that overlapped

Sl.No	Phytoconstituents	*Zingiber officinale* Roscoe	*Cuminum cyminum* L.	*Piper nigrum* L.	*Curcuma longa* L.	*Allium sativum* L.
1.	Beta phellandrene	+	+	-	-	-
2.	1,8-Cineole	+	+	-	+	-
3.	Alpha Curcumene	+	-	-	+	-
4.	Alpha Terpinene	+	-	-	+	-
5.	Alpha-Terpineol	+	-	-	+	-
6.	Alpha Cedrol	+	-	+	-	-
7.	Alpha Copaene	+	-	+	-	-
8.	Linolenic acid	+	-	-	-	+
9.	Alpha Phellandrene	+	+	-	+	-
10.	Alpha Pinene	+	+	-	+	-
11.	Alpha-Selinene	+	+	-	-	-
12.	Beta-Elemene	+	+	+	-	-
13.	Beta-sitosterol	+	+	+	+	-
14.	Kaempferol	+	-	+	-	+
15.	Apigenin	-	+	-	-	+
16.	Beta-bisabolene	-	+	+	+	--
17.	Beta-Carotene	-	+	-	+	-
18.	Campesterol	-	+	+	+	-
19.	Camphene	-	+	-	+	-
20.	Eugenol	-	+	-	+	-
21.	Linalool	-	+	-	+	-
22.	Limonene	-	+	-	+	-

23.	Niacin		+		+	+
24.	Oleic acid	-	+	-	-	+
25.	Citral	-	-	+	-	+
26.	Quercetin	-	-	+	-	+
27.	Caffeic acid	-	-	-	+	+
28.	Alpha tocopherol	-	-	+	+	-
29.	Curcumin	+	-	-	+	-
30.	Vanillic acid	-	-	-	+	+

***'+' = present; '-'= absent**

Table S3: Binding energy scores calculated by iGEMDOCK 2.1 for 153 phytocompounds against CDK8, PR and EGFR target proteins

Sl. No.	Name of the phytocompounds	Target proteins		
		**CDK8** (Kcal/mol)	**PR** (Kcal/mol)	**EGFR** (Kcal/mol)
1	(S)-6-Gingerol	-104.811	-87.502	-79.4371
2	Ajoene	-77.5688	-61.6849	-57.9559
3	Allicin	-57.9275	-53.122	-47.9418
4	Alliin	-79.0241	-62.0727	-59.6716
5	Allixin	-92.5369	-74.6788	-65.9877
6	Allyl methyl disulfide	-41.1782	-33.8852	-33.8556
7	Allyl 2-propenethiosulfinate	-56.8218	-46.6457	-46.7505
8	Alpha-Cedrol	-70.5328	-72.6062	-60.5928
9	Alpha-Copaene	-67.1359	-58.6649	-55.7061
10	Alpha-Cubebene	-75.6275	-65.1254	-63.1143
11	Alpha-Curcumene	-85.0609	-63.6133	-63.0109
12	Alpha-Farnesene	-78.0516	-59.6599	-61.8937
13	Linoleic acid	-100.737	-83.4394	-74.7183
14	Alpha-phellandrene	-57.3464	-46.7841	-48.7804
15	Alpha-pinene	-52.1852	-43.4731	-46.1632
16	Alpha-Selinene	-71.5675	-59.8168	-58.052
17	Alpha-tocopherol	-105.456	-91.8344	-98.4352
18	Alpha-Terpinene	-57.7403	-47.9462	-47.4883
19	Alpha-Terpineol	-57.6343	-49.4668	-47.313
20	Alpha-Thujene	-56.144	-47.0616	-48.4233
21	Alpha-Zingiberene	-78.696	-63.848	-66.4751

22	(+)-Angelicoidenol	-55.4782	-60.2741	-54.1794
23	Anisaldehyde	-58.5142	-49.6015	-46.8539
24	Apigenin	-108.658	-83.1673	-84.6763
25	**Apigetrin**	**-139.527**	**-123.298**	**-105.185**
26	Arachidic acid	-109.896	-87.6715	-70.9994
27	Arachidonic acid	-111.35	-85.6796	-70.0136
28	AR-Tumerone	-86.7863	-77.2265	-68.2934
29	Ascorbic acid	-76.57	-67.4633	-71.5339
30	Auxin	-79.98	-68.76	-66.4178
31	Benzyl cinnamate	-99.3954	-76.6976	-72.5332
32	Beta-bisabolene	-81.1788	-67.1087	-61.7078
33	Beta-Carotene	-82.9778	-88.2893	-73.8559
34	Beta-caryophyllene	-72.4668	-60.059	-52.9973
35	Beta-chlorogenin	-93.6201	-98.4622	-88.2104
36	Beta-Elemene	-71.0103	-60.6097	-58.9066
37	Beta-farnesene	-83.9071	-64.6652	-65.4269
38	Beta-phellandrene	-56.1826	-49.1959	-46.6697
39	Beta-pinene	-49.5787	-41.7821	-46.3293
40	Beta-pinone	-52.7776	-47.2897	-43.6499
41	Beta-selinene	-69.903	-60.197	-53.9192
42	Beta-sesquiphellandrene	-82.1178	-63.5428	-61.1558
43	Beta-sitosterol	-89.7443	-95.3916	-85.3261
44	Beta-terpinene	-58.5214	-49.5633	-46.815
45	Beta-tocopherol	-107.359	-96.1963	-94.4317
46	Biotin	-95.4029	-79.8656	-68.6381

47	Bisabolene	-81.5005	-65.0422	-61.529
48	Bisacumol	-84.4979	-78.2668	-66.2708
49	Bisacurone	-88.3776	-74.1271	-74.2656
50	(+)-Borneol	-51.2928	-47.516	-47.0786
51	Bornyl acetate	-69.0387	-65.1623	-57.0787
52	Caffeic acid	-86.3691	-66.4419	-71.8763
53	Calamene	-76.7772	-65.5655	-53.3784
54	Calebin-A	-129.381	-100.878	-94.3753
55	Campesterol	-92.3234	-93.6405	-84.8295
56	Camphene	-53.4427	-44.5433	-43.2975
57	Camphor	-54.4404	-46.4857	-44.3376
58	Caprylic acid	-69.7488	-62.1456	-54.2471
59	Car-3-ene	-52.8143	-47.463	-44.3274
60	Carveol	-64.5833	-59.9786	-56.6835
61	Caryophyllene	-74.4742	-61.8815	-51.2548
62	Caryophyllene oxide	-69.5605	-62.9912	-52.8001
63	Chlorogenic acid	-131.947	-103.578	-97.3944
64	Choline	-49.9443	-46.7654	-42.3954
65	1,8-Cineole	-51.2648	-46.1637	-46.0929
66	Cinnamic acid	-73.4787	-65.5802	-53.5436
67	Citral	-70.8019	-55.9028	-55.5976
68	Citronellal	-69.9755	-57.7379	-53.4255
69	Citronellal acetate	-91.5807	-72.5183	-67.8081
70	Citrulline	-80.3157	-75.4111	-72.908
71	Cryptone	-60.563	-52.032	-51.7645

72	Cubebin	-120.885	-95.1669	-97.0883
73	Cuminaldehyde	-68.7815	-56.0776	-51.3361
74	Cuminic acid	-75.501	-66.0935	-53.4807
75	Curcumenol	-83.6496	-61.9687	-60.6331
76	**Curcumin**	**-138.149**	-104.672	-92.0263
77	Curcumol	-71.896	-62.702	-60.4566
78	Curdione	-88.4717	-73.0049	-62.0358
79	Curlone	-81.0765	-62.6684	-67.1518
80	Curzerenone	-84.682	-66.6172	-64.787
81	Cyclocurcumin	-103.688	-99.2554	-90.4457
82	**Cynaroside**	**-149.66**	**-118.635**	**-119.18**
83	Delta cadinene	-76.1382	-62.5963	-57.9843
84	Demethoxycurcumin	-135.487	-90.3546	-89.2974
85	Diallyl disulfide	-53.9619	-41.4064	-42.693
86	Allylpropenyl disulfide	-54.8037	-43.6927	-41.5715
87	Endolysin	-70.059	-62.131	-61.8917
88	Epoxydihydrocaryophyllene	-78.5064	-69.4549	-62.2397
89	Eugenol	-73.7118	-67.2077	-67.4335
90	Farnesol	-91.6988	-77.0437	-71.5401
91	Ferulic acid	-84.903	-68.4163	-71.4099
92	4-Gingerol	-114.815	-80.2474	-82.3321
93	GABA	-54.2399	-51.6452	-47.2442
94	Geraniol	-72.9381	-58.9384	-58.5798
95	Geranyl acetate	-78.0023	-65.0232	-68.7127
96	Germacrene	-69.9808	-61.0217	-55.9958

97	[4]-Gingerdiol 3,5-diacetate	-113.397	-94.2363	-90.7951
98	Guaiacol	-66.3319	-57.0343	-57.025
99	Guaiane	-76.2387	-64.8616	-57.7937
100	Heliotropine	-66.8737	-60.3206	-57.6926
101	Hentriacontane	-93.2908	-86.1348	-73.1799
102	Hentriacontanol	-117.809	-96.9005	-79.6905
103	Hentriacontan-16-one	-99.2657	-85.8104	-81.8618
104	Isocaryophyllene	-75.4924	-61.8225	-51.5789
105	Isoeugenol	-77.4084	-65.8263	-63.5447
106	Isopulegol	-64.4883	-55.1258	-53.9236
107	Jasmonic acid	-89.2523	-61.4375	-65.7346
108	Kaempferol	-110.289	-93.179	-90.3149
109	Limonene	-57.8777	-49.3616	-45.6744
110	Limonene-4-ol	-59.8066	-57.2934	-50.5973
111	Linalool	-67.6877	-57.4475	-51.9731
112	Linalyl acetate	-80.4871	-68.0118	-58.5329
113	Linolenic acid	-118.295	-92.2788	-74.7183
114	Luteolin	-108.428	-87.536	-92.6124
115	Methyl caffeic acid	-92.8714	-68.8333	-73.6634
116	Methyl carvacrol	-65.5959	-61.5457	-54.0494
117	Methyl cinnamate	-80.11	-58.8759	-58.1837
118	24-methylenecycloartanol	-107.072	-100.311	-99.4798
119	Monoterpenes	-70.6298	-51.8586	-53.7995
120	Myrcene	-61.2095	-52.464	-48.5424
121	Myrisitic acid	-103.862	-76.2692	-64.9643

122	Myristicin	-78.1526	-67.2257	-57.1693
123	Myrtenol	-60.9135	-56.8281	-53.0415
124	N-formylpiperidine	-53.2442	-44.8131	-45.3401
125	Niacin	-66.5919	-51.1668	-53.8382
126	Oleanolic acid	-109.046	-99.1252	-75.828
127	Oleic acid	-106.257	-88.9538	-75.5984
128	Pantothenic acid	-88.2894	-80.2365	-75.9183
129	P-cymene	-57.1533	-49.0912	-47.3159
130	Pectin	-83.04	-69.6256	-67.4675
131	Piperitol	-106.28	-90.9885	-96.8025
132	Piperitone	-61.9344	-54.3268	-96.8025
133	Quercetin	-113.226	-86.9838	-91.5963
134	Riboflavin	-129.65	-104.237	-100.923
135	Salicyclic acid	-74.8269	-64.1135	-60.2876
136	Sesquisabinene	-82.8085	-62.5643	-61.1352
137	6-Gingerol	-99.1522	-87.4194	-84.6827
138	Stigmasterol	-102.506	-112.951	-90.5877
139	Succinic acid	-70.6602	-56.2248	-49.2229
140	Allyl methyl thiosulfinate	-53.39	-49.8601	-44.7704
141	10-Epizonarene	-78.6916	-62.7661	-53.7485
142	10-Gingerdione	-123.636	-92.2556	-91.8284
143	10-shogaol	-122.044	-92.496	-91.4592
144	Terpinen-4-ol	-60.5644	-51.5869	-49.8435
145	Thymol	-64.2775	-55.8671	-58.7873
146	Allyl methyl trisulfide	-48.1138	-38.3366	-37.7196

147	Trigonelline	-66.0554	-52.8032	-56.3241
148	12-Gingediol	-110.973	-91.7979	-87.4242
149	12-Gingerol	-120.553	-97.8895	-87.0361
150	2-vinyl-1,3-dithiine	-47.2528	-41.8305	-42.4637
151	Vanillic acid	-77.6887	-69.4284	-69.8352
152	Vinyldithiin	-49.5499	-42.1021	-41.6892
153	**Xyloglucan**	-111.862	**-113.788**	**-105.106**
